# The Fluid Percussion Injury Model of Traumatic Brain Injury: Insights into Post-Traumatic Epilepsy and Comorbidities

**DOI:** 10.3390/ijms27209127

**Published:** 2026-10-15

**Authors:** Jaclyn Iannucci, Anna Sentmanat, Kelly Dickerson, Maryam Obaid, Shreya Bandopadhyay, Ann Houghton, Gabriel Maisonnaive Arisi, Lee A. Shapiro

**Affiliations:** Department of Neuroscience and Experimental Therapeutics, Texas A&M Health Science Center, Bryan, TX 77807, USA; jmiannucci@tamu.edu (J.I.); acsent@tamu.edu (A.S.); kdickerson@tamu.edu (K.D.); mobaid@tamu.edu (M.O.); shreyabandopadhyay@tamu.edu (S.B.); ann.houghton@tamu.edu (A.H.); garisi@tamu.edu (G.M.A.)

**Keywords:** seizure, comorbidity, epileptogenesis, concussion, rodent, electrophysiology

## Abstract

Traumatic brain injury (TBI) affects millions of people each year and can lead to persistent neurological disorders, including post-traumatic epilepsy (PTE). Animal models of concussion and TBI, developed since the late nineteenth century, provide a means to investigate injury progression and its consequences under controlled conditions. The lateral fluid percussion injury (LFPI) model is widely used to study both TBI and post-traumatic epileptogenesis. PTE is often resistant to antiseizure medications, and variability among experimental models (e.g., strain, injury, surgical, and physiological differences) remains a major obstacle to developing antiepileptogenic treatments. Understanding the factors that influence model reproducibility is therefore essential. This review first examines the development of the LFPI model and the experimental factors that influence PTE outcomes. It then considers the cellular mechanisms of post-injury hyperexcitability and the affective, cognitive, and neurodegenerative changes associated with LFPI. Together, these findings indicate the value of the model while defining the limits of its application to human disease.

## 1. Introduction

### 1.1. Traumatic Brain Injury

Traumatic brain injury (TBI) affects an estimated 2–3 million people annually in the United States (US) and more than 50 million worldwide [1,2]. More than 20 million Americans over the age of 40 have reported a TBI involving loss of consciousness [3]. Long-term complications include pain, depression, cognitive impairment, post-traumatic epilepsy (PTE), and an increased risk of neurodegenerative disorders such as Alzheimer’s disease (AD) and Parkinson’s disease (PD) [4,5,6,7,8,9]. Despite this burden, biomarkers and treatments for many post-traumatic syndromes remain limited.

TBI is defined as a change in normal brain function caused by an external force. Manifestations include altered consciousness, memory loss, changes in mental state, and neurological deficits; severe injuries can result in coma, a vegetative state, or brain death. Injuries may be closed or penetrating. In the cited study of armed forces service members, 38% of injuries were closed, and 62% were penetrating [10]. TBI severity ranges from mild to severe. Mild TBI, including concussion, has been described as an alteration in brain function lasting 24 h or less, with or without loss of consciousness and a Glasgow Coma Scale (GCS) score of 13–15. Concussions and other mild injuries account for approximately 75–85% of TBIs. Moderate TBI has been classified as altered brain function lasting more than 24 h, with loss of consciousness, amnesia lasting up to 7 days, and a GCS score of 9–12; it accounts for approximately 10% of TBIs. Severe TBI, which accounts for approximately 2–3% of cases, is characterized by prolonged unconsciousness and amnesia, with a GCS score below 9 [11,12].

Animal models are essential for investigating post-injury pathology and the mechanisms underlying post-traumatic disorders. Experimental studies of concussion date to at least 1878 [13], and early approaches informed the development of the models used today [14,15,16,17,18]. These include controlled cortical impact (CCI), fluid percussion injury (FPI), weight-drop injury, and blast injury. FPI is widely represented in the literature. This review traces the development of the FPI model and examines its applications to PTE and associated comorbidities.

### 1.2. The Fluid Percussion Injury (FPI) Model

Denny-Brown and Russell introduced an early precursor of the FPI device in 1941 [19], and their work continues to be cited in later studies [20]. They modeled concussion by delivering a fluid pulse to the exposed dura of cats. The pulse altered cerebral blood flow and blood pressure and, in some cases, resulted in death [19]. Lindgren and Rinder subsequently conducted a systematic series of studies in rabbits. Their plunger system rapidly displaced a fluid column onto the exposed dura, applying a mechanical load to the brain. This approach foreshadowed the modern FPI device [21,22,23,24].

Sullivan and colleagues first used the modern FPI device (Figure 1) in 1976 [25]. The device delivered a single, brief hydraulic pressure pulse to the exposed dura of cats [25], producing elastic brain deformation resembling that observed in clinical head injury. Physiological and pathological responses correlated with peak pressure such that higher pressures produced larger lesions and more extensive subarachnoid hemorrhage [25]. Electroencephalographic (EEG) changes were found to correlate with neuropathological alterations but appeared only after high-pressure injury. The model was later adapted for rats [26,27], reproducing pressure-dependent pathology and allowing investigators to assess apnea and changes in blood pressure.

Along with pressure, injury location also influences the resulting pathology. At mild to moderate pressures of approximately 1.2–2.5 atmospheres (atm), midline FPI typically produces diffuse axonal injury without a substantial focal lesion [28,29]. In contrast, lateral FPI (LFPI) produces both focal and diffuse injury [30,31,32,33]. Studies in rats [30,31] and mice [34] show that the extent of neuropathology and neurobehavioral impairment depends largely on injury location and severity.

## 2. Modeling Acquired Epilepsy Using the LFPI Model of Traumatic Brain Injury

Clinical TBI can cause motor, affective, and cognitive impairments, as well as seizures. PTE occurs in approximately 5–7% of patients, although estimates vary considerably among studies, compared with an epilepsy prevalence of 0.5–2% in the general population [35,36,37]. The factors that determine individual risk remain unclear, but studies indicate that greater injury severity is associated with a higher likelihood of PTE. Consistent with this notion, rates as high as 50% have been reported after severe TBI [35,38,39,40]. Recent analyses of the TBI Model Systems cohort have developed individualized models of PTE risk at 1 and 2 years after moderate-to-severe TBI, providing a clinical framework for evaluating candidate prognostic markers [41]. PTE is frequently resistant to first- and second-line antiseizure medications [42,43,44], with veterans representing the highest risk [45]. Preclinical FPI studies likewise show substantial variation in epilepsy outcomes [46]. Examining their methods and findings can help identify the sources of this variability and highlight potential pathological mechanisms in PTE.

D’Ambrosio and colleagues characterized rat LFPI as a model of PTE [47,48,49]. In their initial studies, a single severe injury at 3.75–4 atm induced chronic epileptiform activity in 92% of rats [47], and spontaneous seizures increased over time [48]. Another study found focal seizures in 50% of rats after LFPI at 3.2–3.5 atm [50]. Repetitive high-frequency oscillations with spikes also increased over the following year, alongside increases in focal seizure frequency and duration [50]. Other studies have reported lower rates of spontaneous seizures. Kharatishvili and colleagues found that 43–50% of rats developed spontaneous seizures by 12–13 months after LFPI at 2.6–3.3 atm [51,52]. Shultz and colleagues [53] and Liu and colleagues [54] reported spontaneous seizures and/or epileptic discharges in over 50% of rats within 6 months of severe LFPI at 3.2–3.5 atm. Medel-Matus and colleagues detected spontaneous seizures in 30% of rats at 7–9 months after a 2.0 atm injury, with spontaneous seizure occurrence associated with faster kindling [55].

Injury severity and location may partly explain these reported differences. In one study, higher neurological severity scores (NSS) were associated with more frequent seizures [56]. In another, behavioral impairment did not predict spontaneous seizures, whereas weight loss during the first post-injury week provided some prognostic information [57]. These findings suggest that injury severity contributes to seizure outcomes but does not fully explain individual susceptibility.

As in clinical PTE, additional factors influence whether and when spontaneous seizures develop. Differences in experimental methods may also contribute to variability in seizure response among studies. Relevant parameters include injury location, animal age, sex, and strain, burr hole size, needle and syringe assembly, injury severity, EEG recording sites and schedules, and the duration of follow-up [47,48,49,50,51,52,58,59], as shown in Table 1. Brain abscesses associated with cranial surgery and electrode implantation can provide an alternative seizure etiology in LFPI studies, supporting systematic histological assessment when interpreting post-traumatic seizures. In addition, the method of skull closure can be variable and is inconsistently reported across studies. Considering that closing the cranial window after LFPI may alter intracranial pressure, this too should be considered in LFPI studies [60,61]. Nevertheless, LFPI provides a useful model for investigating post-traumatic epileptogenesis, particularly when experimental variables are carefully controlled.

Although rats are used more often, mice also provide a model for studying PTE after LFPI. After a moderate to severe injury at 2.9 atm (3 mm craniotomy over left parietal), 71% of mice exhibited spontaneous epileptiform spiking and 58% exhibited epileptiform discharges, but late spontaneous EEG seizures were detected in only 3% [62]. Studies using genetically diverse recombinant inbred Collaborative Cross (CC) mice have identified strains with greater susceptibility to PTE following LFPI [63,64]. In particular, more than 80% of mice from the CC031 strain developed post-traumatic seizures after mild to moderate LFPI at 1.2–1.5 atm (3 mm craniotomy over right parietal), with seizures progressing over time [63]. PTE development was associated with dynamic high-amplitude rhythmic spikes (DHRS) during the first 12 h after injury. Increased delta power in the acute phase and altered delta/gamma coupling also predicted later PTE [64]. These findings suggest that genetic background contributes substantially to seizure outcomes and that a broader range of mouse strains may improve the translational relevance of LFPI studies for PTE.

## 3. Hyperexcitability and Increased Seizure Susceptibility After LFPI

In addition to directly modeling PTE, LFPI can produce persistent hyperexcitability and increased seizure susceptibility even when spontaneous seizures are not detected [65,66]. This susceptibility is often assessed using a second challenge after injury. In rats, LFPI increases vulnerability to pentylenetetrazole (PTZ)-induced seizures [56,58,67,68,69]. For example, a subconvulsant PTZ dose given 5 weeks after LFPI shortened the latency to the first clonic seizure, prolonged the duration of generalized clonic seizures, and increased EEG wave amplitude [70]. Notably, susceptibility was greater in rats with higher NSS values [56]. Increased seizure susceptibility has also been reported after kainic acid (KA) administration [71]. Peripheral immune challenges, including lipopolysaccharide (LPS), can further enhance post-LFPI seizure susceptibility, including responses to PTZ [72,73]. Mice show similar increases in susceptibility after LFPI [62], with greater PTZ-induced seizure severity, frequency, and duration during the first 30 days after injury [66]. Several CC strains that did not develop spontaneous PTE nevertheless showed increased PTZ susceptibility [63]. Thus, provoked seizure susceptibility and spontaneous epilepsy are related but distinct outcomes.

The hippocampal dentate gyrus (DG) is a major site of post-LFPI hyperexcitability. Injury disrupts the balance of excitatory and inhibitory synaptic transmission onto dentate granule cells [32,33,74,75,76,77,78,79,80,81], which subsequently show more action potential firing and longer depolarizations in response to perforant path stimulation [77,82]. Damage to parvalbumin-expressing interneurons (PV-INs) and reduced tonic GABA currents further weaken inhibition [75,79,83]. Dentate hyperexcitability correlates with hilar neuron loss [32] and may enhance signaling to CA3 [79], whereas synaptic efficacy in CA1 can decrease [76]. Hyperexcitability is present bilaterally at 7 days post-LFPI but persists chronically only on the ipsilateral side [80]. Beyond the DG, LFPI can increase the excitability of CA1 pyramidal neurons [84]. Loss of synaptic inputs and impaired CA1 synchronization may also disrupt inhibitory modulation of the medial septum, a key generator of hippocampal theta oscillations [85]. These changes provide several potential routes from injury to hippocampal network dysfunction.

LFPI also alters DG neurogenesis, producing an acute increase followed by a persistent decrease [86,87,88,89], together with abnormal granule cell growth and connectivity, using the hypertrophic radial glia as a scaffold [90,91,92]. These alterations to hippocampal neurogenesis are associated with increased neuronal excitability, seizure susceptibility [86,87,88,89,93], and epilepsy development [94]. Abnormal growth, migration, and integration of newborn neurons can contribute to hyperexcitable circuits [94,95,96,97,98,99,100]. In seizure models, neurons generated after seizures can develop altered morphology [101], migrate ectopically into the hilus, extend hilar basal dendrites, and exhibit aberrant mossy fiber sprouting [96,99,102]. Pharmacological targeting of the acute neurogenic increase has prevented increases in dentate excitability and seizure susceptibility [93].

Seizures also promote the functional integration of adult-generated granule cells [99]. Granule cell mossy fibers have been shown to grow along an ectopic glial scaffold [103] and form recurrent synapses onto the aberrantly sprouted granule cell hilar basal dendrites [95]. Ectopically integrated granule cells can remain stable, receive increased mossy fiber input, and form recurrent excitatory circuits that lower seizure thresholds [104,105,106,107,108,109]. These findings suggest that seizure-induced newborn neurons can help sustain pro-epileptogenic circuitry [110,111]. Similar disturbances of neurogenesis and circuit remodeling after LFPI may contribute to post-traumatic hyperexcitability, although increased seizure susceptibility does not necessarily indicate spontaneous PTE. It is pertinent to note that large differences have been reported between adult neurogenesis in laboratory settings and humans, for which studies are highly variable, with some continuing to maintain that human adult hippocampal neurogenesis is marginal at best [112,113,114]. These discrepancies highlight the translational uncertainty of targeting rodent-based mechanisms to treat human PTE.

Reduced expression of GABA_A_ receptor subunits has been reported in human epilepsy tissue [115]. In rats, LFPI alters GABA_A_ receptor subunit expression and causes PV-IN loss in the ipsilateral thalamus and hippocampus [68,116], potentially increasing neuronal excitability [116]. The reduction in hippocampal GABA_A_ subunits was bilateral and transient, whereas thalamic reductions were ipsilateral and persistent. GABA_B1_ and GABA_B2_ subunits were also downregulated [116]. Loss of parvalbumin-positive GABAergic thalamic neurons after LFPI has been associated with greater seizure susceptibility [68]. Together, these changes may weaken the inhibition that normally constrains seizure activity. Ceftriaxone provides one example of an intervention targeting the balance between excitation and inhibition. By increasing GLT-1 expression, ceftriaxone may improve glutamate clearance and attenuate the loss of cortical inhibition. In rats treated with ceftriaxone after LFPI, cortical inhibition returned to near-baseline levels within 4 weeks [117]. GLT-1 expression increased during the 1-week treatment period but returned to baseline 6 weeks after treatment ended [117]. These data indicate that the loss of inhibitory interneurons after FPI may promote a pro-epileptic state.

## 4. Glial, Inflammatory, and Metabolic Mechanisms of Epileptogenesis After FPI

Neuronal excitability after LFPI is also influenced by glial activation, inflammatory signaling, and mitochondrial function. Astrocytes and microglia are central to the brain’s inflammatory response after LFPI [4,118,119]. Their activation begins rapidly and generally peaks at 3–7 days, although the time course depends on injury severity, location, and species [66,87,119]. Low-level activation can persist chronically [119]. In a mouse LFPI model that incorporated a second-hit seizure challenge, astrocytes were observed to migrate to the injury site by day 1, with pronounced peri-injury activation at day 3 that began to subside by day 7 [66]. Iba1+ microglia, including rod microglia, likewise increased in the ipsilateral cortex 3 days post-LFPI and began to decline by day 7 [66].

### 4.1. Astrocytes and Associated Mechanisms in LFPI

Astrocytes can directly influence epileptogenesis [120]. Persistent astrocyte activation in human epileptic foci and changes to astrocytic inwardly rectifying potassium currents may impair extracellular ion regulation and promote hyperexcitability [121]. Loss of astrocyte gap junction coupling can disrupt ion buffering and is associated with altered calcium signaling and glutamate release [122]. Furthermore, increased astrocytic glutamate release can lower the seizure threshold [122]. Astrocytes also release ATP as a gliotransmitter, which activates purinergic P2 receptors [122]. Clinically, extracellular ATP is elevated in patients with drug-resistant epilepsy, potentially increasing P2 receptor activation [122]. Although the contribution of this pathway remains incompletely understood, P2 receptor blockade has shown anticonvulsant and neuroprotective effects [122].

Astrocytes also regulate neurotransmitter clearance, a process disrupted after FPI [4,123]. Excitatory amino acid transporters (EAATs) normally remove glutamate from the extracellular space [124]. TBI can alter the expression and function of glutamate transporters, including GLAST and GLT-1 in rodents [125]. Reduced uptake elevates extracellular glutamate and can increase excitability and seizure susceptibility [117,118,126]. FPI also triggers an acute glutamate surge that contributes to excitotoxicity and sustained microglial inflammatory responses [118,127].

Toll-like receptor 4 (TLR4) signaling provides another link between injury and disrupted glutamate signaling [128]. Neuronal TLR4 responds to damage-associated molecular patterns (DAMPs) released after injury. The resulting enhanced signaling increases GluA1 surface expression and calcium-permeable α-amino-3-hydroxy-5-methyl-4-isoxazolepropionic acid receptor (AMPAR) currents, promoting dentate granule cell excitability [128]. Furthermore, excess glutamate is also associated with altered expression of immediate early genes (IEGs), including the proapoptotic factor Bax, brain-derived neurotrophic factor (BDNF), and the activity marker c-fos [129]. These expression changes may indicate hippocampal injury, but their value as PTE biomarkers requires validation. Additionally, N-methyl-D-aspartate receptor (NMDAR) antagonism reduces Bax and BDNF expression after FPI [129], supporting further investigation of this pathway as an acute therapeutic target.

Glial changes may also alter the structural environment in which newborn neurons develop. GFAP+ radial glia in the DG provide a scaffold for newborn neurons to migrate [103]. LFPI can induce changes to the radial glial-like astrocytes that favor abnormal neuronal growth, migration, and development [90,92]. The resulting circuitry may support recurrent excitation among granule cells, facilitate seizure spread [4], and contribute to epileptogenesis [111]. Although hilar basal dendrites are usually transient in normal rodent granule cells, they are more common in nonhuman primates and humans [130,131,132]. Their extension along an ectopic glial scaffold may therefore have translational relevance even while the extent of functional adult hippocampal neurogenesis in humans remains debated.

### 4.2. Microglial Mechanisms in LFPI

Microgliosis is well documented after LFPI [133,134,135], and persistent microglia-driven proinflammatory responses may contribute to PTE [118,136,137]. In rats, microglial density correlated with epileptiform spike incidence after LFPI [138], while the selectively bred SLOW rat strain, which has lower susceptibility to acquired epilepsy, showed reduced microglial activation after injury [59]. Conversely, CC031 mice, which developed PTE after moderate LFPI, showed the highest cortical Iba1 staining among the strains examined after injury [63]. These associations suggest that microglial responses are related to post-traumatic hyperexcitability, although they do not establish a causal role.

Microglia may influence this hyperexcitability through their effects on post-injury neurogenesis and neuronal integration [139,140,141]. Altered synaptic pruning offers another possible mechanism [142], as excessive or aberrant removal of synapses may disturb the balance between excitation and inhibition and aggravate epileptogenic processes [142]. Notably, microglia exhibit acute increases in phagocytic capacity after LFPI [135]. Furthermore, bulk and single-nucleus RNA sequencing identified inflammatory transcriptional changes in the dentate gyrus 3 weeks after rat LFPI, with contributions from microglial and astrocytic populations [143]. These observations support further investigation of astrocyte–microglia interactions in post-traumatic epileptogenesis.

Activated astrocytes and microglia release cytokines and chemokines that can alter neuronal excitability. Interleukin (IL)-1β, tumor necrosis factor-α (TNF-α), and IL-12 participate in inflammatory signaling that can sustain immune activation through the generation of inflammasomes [118,127]. FPI increases proinflammatory cytokine release, consistent with reports of elevated IL-1β, high mobility group box 1 (HMGB1), and TNF-α in human epileptic foci [144]. Moreover, TLR4 activation and IL-1β signaling have been associated with increased excitability in hippocampal CA1 neurons [144,145]. TNF-α can also increase synaptic AMPAR clustering, strengthening excitatory transmission and potentially promoting excitotoxicity [144]. Cytokines can further alter excitatory output, inhibitory signaling [118], synaptic strength, and plasticity, providing additional routes by which inflammation may influence seizure development [118].

### 4.3. Metabolic and Vascular Components of LFPI

Cellular stress and metabolic dysfunction may also contribute to epileptogenesis after LFPI. Endoplasmic reticulum (ER) stress arises when misfolded proteins accumulate and can activate proapoptotic pathways [146]. After LFPI, increases in ER stress-related proteins, including PERK, eIF2α, ATF4, and GRP78, indicate activation of the unfolded protein response (UPR) pathway [146]. Sustained ER stress can promote cytochrome c release and reduce antiapoptotic proteins such as Bcl-2. This can potentially contribute to neuroinflammation and reactive oxygen species (ROS) production seen in PTE. To that end, pretreatment with an ER stress inhibitor, Salubrinal, decreased activation of UPR pathways following TBI [146]. Mitochondrial dysfunction may likewise increase ROS production and disrupt calcium homeostasis, neuronal excitability, and synaptic transmission [147]. After LFPI, mitochondrial dysfunction correlates with injury severity [148] and includes altered expression of electron transport chain (ETC) subunits [149], reduced mitochondrial protein content, and lower ATP production [150].

LFPI also damages the blood–brain barrier (BBB), disrupts vascular function, and increases vascular permeability, which promotes inflammation and seizure susceptibility [151,152,153,154]. Blood-based markers of vascular injury, including von Willebrand factor (vWF), have been investigated in relation to post-traumatic seizure susceptibility [155]. Macrophage migration inhibitory factor (MIF), an inflammatory cytokine elevated after LFPI, has also been associated with increased BBB permeability [119,156,157]. Furthermore, barrier disruption facilitates the entry of peripheral immune cells into the central nervous system (CNS) [158], which can contribute to the overall pro-inflammatory environment after LFPI. In addition, hemorrhage can leave iron and hemosiderin deposits within the brain tissue, promoting oxidative and nitrosative stress. These processes have been linked to neurotoxic guanidino compounds with endogenous convulsant activity [126]. Albumin leakage into the parenchyma can also disturb potassium regulation and increase neuronal excitability, contributing to PTE development [159,160]. BBB dysfunction therefore interacts with glial, inflammatory, and metabolic changes that may contribute to epileptogenesis after FPI.

## 5. Neurobehavioral Effects of LFPI

LFPI produces short- and long-term affective and cognitive changes that resemble aspects of clinical TBI. Depression and anxiety have been reported in up to 70% of patients after TBI [161,162,163,164,165,166], and estimates of post-injury cognitive impairment range from 50% to 70% [167,168,169,170,171]. Whether the severity of these deficits predicts PTE remains unclear. Nevertheless, affective and cognitive disorders are also common in people with epilepsy [172,173,174,175], making these outcomes relevant to studies of post-traumatic comorbidity.

### 5.1. Negative Affect After LFPI

Depression and anxiety are common after TBI and can impede patient recovery and rehabilitation. Reported anxiety rates reach up to 70%, and depressive disorders affect approximately 25–50% of TBI survivors [176]. Depression is generally most frequent during the first year post-injury and subsequently declines or remains stable [161,162,163,164,165,166,176,177]. However, some patients initially presenting with minor depression symptoms later develop major depressive disorder (MDD) [162], and clinically significant anxiety may increase over time [178]. Risk factors for post-injury depression and anxiety include gender, pre-injury history of psychiatric illness, and unemployment [164]. Patients with post-TBI anxiety and depression also report greater injury severity and worse cognitive functioning [177]. Understanding how these disorders develop is important for improving quality of life, social functioning, and rehabilitation outcomes after TBI.

LFPI studies reveal considerable variation in affective outcomes across injury conditions and affective-associated behavioral tests. In mice, moderate LFPI at approximately 0.83 atm produced progressive depression-like behavior measured by the tail suspension test beginning 6 months after injury [179]. In rats, mild LFPI at approximately 1.2 atm transiently reduced anxiety-like behavior in the elevated plus maze (EPM) but did not produce long-term depression-like deficits in the forced swim test (FST) [180]. In contrast, da Silva Fiorin and colleagues reported reduced sucrose consumption and social interaction after mild rat LFPI at 1.1–1.4 atm. Increased open-arm exploration in the EPM was interpreted as impulsivity, without significant anxiety-like behavior [181]. Severe rat LFPI at approximately 3.5 atm produced anxiety-like behavior in the open field and EPM for up to 6 months [182]. These differences suggest that injury severity contributes to affective outcomes, although test selection and follow-up duration also shape their interpretation. Repeated injury can further modify affective outcomes. Shultz and colleagues found that more frequent mild LFPI at 1.0–1.5 atm increased anxiety- and depression-like behavior in rats [183]. Tan and colleagues similarly reported increased depression-like behavior in the FST after three mild injuries, but reduced anxiety-like behavior [184]. Another study found that treatment to reduce neuroinflammation after LFPI alleviated anxiety-like behavior and cognitive impairment after repeated injury [185].

Inflammatory challenges may also worsen affective outcomes after a single injury. Fenn and colleagues linked immune activation and microglial priming to depression-like behavior 1 month after diffuse injury induced by midline FPI [186]. Hyperbaric oxygen administered immediately after LFPI reduced hippocampal CA3 microglial activation and TNF-α expression and attenuated depression-like behavior by day 15 in rats [187]. These findings suggest that injury severity, repetition, and neuroinflammation jointly influence affective outcomes in LFP and provide a basis for testing targeted interventions.

### 5.2. Cognitive Impairment After LFPI

Cognitive impairment is a common and sometimes persistent consequence of TBI [167]. Clinically, deficits in attention, memory, and executive function [168] can reduce quality of life and increase care needs [169,170]. Animal studies assess related functions using behavioral tasks designed to probe specific cognitive domains [188]. Across FPI studies, cognitive impairment, including hippocampus-dependent deficits, are common, although their severity and duration vary with injury parameters and assessment timing. Lifshitz and colleagues reported impaired conditioned fear responses 7 days after midline FPI, with deficits in memory consolidation rather than acquisition or recall [189]. Performance recovered by 1 month, indicating that some cognitive effects can be transient. Spain and colleagues found impaired spatial learning and memory in the Morris water maze (MWM) after mild mouse LFPI at approximately 0.9 atm [190]. Gurkoff and colleagues extended these assessments to metric, topological, and temporal ordering memory in rats [191]. Different memory domains were affected to different degrees, and greater injury severity was associated with greater impairment. These studies show the value of assessing multiple cognitive functions after injury.

Age, sex, injury severity, and injury location can influence cognitive outcomes. After moderate LFPI at 1.2 atm to the right parietal lobe of C57BL/6 mice, memory deficits differed by sex [192]. Paired associate learning (PAL), Barnes maze, and fear conditioning tests at 7 and17 weeks post-injury showed greater vulnerability to anterograde memory impairment in males, while retrograde memory remained intact. In a juvenile LFPI model using 21-day-old C57BL/6J mice, males showed lower acute inflammatory cytokine expression but greater subacute microglial/macrophage accumulation and greater neurological recovery than females [193]. Recent mouse LFPI experiments found sex-dependent changes in the hippocampal neurogenic niche and greater pattern recognition deficits in females; antagonism of class II-associated invariant peptide (CLIP) improved cognitive performance, supporting further study of immune contributions to these differences [91]. Injury laterality may also matter, as both left and right LFPI impaired MWM performance in C57BL/6J mice, but right-sided injury produced a slightly slower acquisition rate [194].

Interestingly, early injury severity measures do not always predict chronic cognitive impairment. In adult male Sprague–Dawley rats, righting reflex time, neurological severity scores, weight loss, and open field performance correlated poorly with T-maze performance 60 days after LFPI [195]. Cognitive deficits can also occur without substantial neuronal loss. Rats injured on postnatal day 19 showed impaired MWM performance despite no significant neuronal loss in the cortex or hippocampal CA3 region [196]. Similarly, mild LFPI at 1.0–1.5 atm impaired spatial learning and short-term memory in male Sprague–Dawley rats without visible neuronal loss, although inflammation and axonal injury were present [197]. That study combined behavioral testing with diffusion tensor imaging (DTI), immunohistochemistry, and silver staining to assess functional and structural outcomes. These observations indicate that the timing of assessment, injury severity and location, age, and sex should be considered when interpreting cognitive outcomes after FPI.

## 6. Neurodegenerative Disorders with Seizure Components That Are Facilitated by TBI, and Modeled by LFPI

### Alzheimer’s Disease

AD, the most common type of dementia, is a progressive neurodegenerative disease characterized by protein aggregation and neuronal loss, particularly in the hippocampus and cerebral neocortex [198]. The two pathological hallmarks of AD are aggregated extracellular amyloid-β (Aβ) plaques and intracellular neurofibrillary tangles composed of hyperphosphorylated tau [199]. As the disease progresses, memory, cognition, behavior, and everyday functioning decline. TBI is associated with an increased risk of AD and related dementias (ADRD), with risk varying by injury severity and repetition [200]. A history of TBI has also been associated with an earlier age of AD onset [5,201,202]. While AD and TBI are two devastating neurological disorders with overlapping clinical manifestations, their complex relationship is not completely understood. Therefore, combining the LFPI model with transgenic AD models offers a means to investigate injury-induced changes relevant to this association [151]. Epilepsy has been reported in 10–22% of people with AD [203], and seizures are more frequent in people with AD and other dementias than in older adults without dementia [204,205]. Shared mechanisms may include excitotoxicity, inflammatory signaling, and tau- and Aβ-associated pathology [206].

Tau pathology may link epilepsy and AD pathogenesis [207]. In a study of adults younger than 60 years with mesial temporal lobe epilepsy (MTLE), phosphorylated tau (p-tau) expression was elevated in sclerotic hippocampi compared with controls [208]. Neuronal, glial, and extracellular p-tau deposits were detected in 95% of patients in brain regions associated with epileptic activity [208], and neurofibrillary tangles were present in pyramidal neurons in 67% of patients, and neuronal pre-tangles were observed in all patients [208]. Greater p-tau burden was associated with higher seizure frequency and attention deficits, suggesting a possible contribution to cognitive impairment in MTLE. Consistent with the clinical data, increased tau phosphorylation and accumulation have also been observed after FPI [209]. Tau supports microtubule stability and axonal transport. Although tau phosphorylation regulates its normal function, excessive phosphorylation can destabilize the cytoskeleton [210], impair transport, and contribute to synaptic loss and neuronal death. Tau-associated synaptic dysfunction may increase neuronal excitability and lower seizure thresholds [211]. Moreover, pathological tau aggregates can also spread across brain regions involved in seizure generation and propagation. Before neurofibrillary tangles form, soluble tau species may enhance glutamatergic activity and alter network organization, increasing interictal epileptiform discharges (IEDs) and electroclinical seizures in AD models [212]. These findings provide a potential mechanistic link between tau pathology and epileptogenesis.

LFPI may also influence Aβ accumulation [151]. Aβ can form soluble oligomers and insoluble deposits that disrupt cellular function. FPI studies have linked increased Aβ deposition in the brain to axonal damage and neuronal loss [151,213], and persistent axonal Aβ accumulation has been reported after brain trauma [214]. Aβ can disturb the balance between excitation and inhibition in cortical and hippocampal networks [215]. Increased neuronal activity may precede plaque formation and can be accompanied by disrupted slow-wave oscillations and increased network synchronization [215].

Aβ-related dysfunction involves glutamate receptors, calcium signaling, and mitochondria [211]. Aβ oligomers can activate NMDARs, promoting calcium influx and neuronal injury [216]. Aβ also disrupts calcium/calmodulin-dependent protein kinase II (CaMKII) signaling and AMPAR trafficking, altering receptor distribution and long-term potentiation (LTP) [217]. Reduced AMPAR expression can weaken synaptic transmission and plasticity [218]. Another proposed mechanism by which Aβ promotes excitability involves ER stress associated with elevated intracellular calcium and proapoptotic signaling [211]. ER stress may promote further Aβ formation, while Aβ-related inflammation and caspase-3 activation can amplify cellular injury [211]. Aβ-associated tau cleavage and mitochondrial dysfunction may further increase tau toxicity and abnormal phosphorylation [211]. These interacting pathways suggest how injury-related Aβ and tau changes could contribute to seizure susceptibility.

Neuroinflammation provides another possible link among TBI, AD, and epilepsy. Inflammasomes are multiprotein complexes that promote IL-1β and IL-18 release and pyroptotic cell death. NLRP3 and related inflammasome pathways have been implicated in AD and TBI models [219,220]. After FPI, DAMPs released by injured cells can activate these pathways [221,222]. Their products stimulate microglia and astrocytes, which can interact with AD-associated proteins and amplify pathology. Genetic studies of triggering receptor expressed on myeloid cells 2 (TREM2) further implicate innate immunity in AD [223]. TREM2 is expressed by myeloid cells, including microglia and macrophages, and certain variants are associated with increased AD risk [223]. In an LFPI study, TREM2 expression increased after injury. TREM2-deficient mice showed altered macrophage distribution acutely and reduced hippocampal atrophy and behavioral impairment at 120 days post-injury compared with injured wild-type mice [224]. Peripheral immune recruitment may also affect post-traumatic pathology. CCL2 signaling through its receptor CCR2 helps regulate monocyte entry into the brain after TBI [225], and CCR2-associated macrophage responses are increased after injury [226,227,228]. In the LFPI model, CCR2 deletion reduced lesion volume and axonal pathology but increased p-tau levels and altered its localization in the hippocampus and cortex [225]. This divergence indicates that reducing one component of post-injury inflammation does not necessarily improve every pathological outcome.

## 7. Future Perspectives and Additional Considerations

### 7.1. Translational Failures Require a Search for Novel Epileptogenic Mechanisms

The vast majority of PTE-relevant lab discoveries either never leave the lab or fail to translate. There are many reasons for this, including poor model appropriateness, underpowered or faulty experimental designs, and lack of consideration of sex as a biological variable. While the number of anti-epileptic drugs has increased, the percent of drug resistance in PTE remains unchanged. As indicated above, PTE is frequently resistant to first- and second-line antiseizure medications [42,43,44]. Therefore, novel targets and mechanisms need to be explored to provide better treatment options for the ~20 to 45 percent of PTE patients who are refractory to anti-epileptic drugs [43,229]. One such potential target is the gastrointestinal (GI) system [230].

Studies in TBI clearly demonstrate gut dysfunction following injury [231,232,233], including gut dysmorphia and blood-gut barrier breakdown by three days post-LFPI [234]. These studies highlight a bi-directional link between the brain and gut, allowing intestinal inflammation to migrate to the brain [235,236]. As neuroinflammation has been identified as a pathophysiological cause of seizures [237], and given the finding that intestinal inflammation impacts the efficacy of anti-convulsant drugs [238], the intestines may represent a novel target organ for addressing the notable resistance of PTE to anti-epileptic drugs.

Furthermore, because the blood-gut barrier has been demonstrated to be leaky after TBI [234], potentially pro-epileptogenic substances such as LPS, can not only leach into the blood, but they can also exacerbate BBB breakdown, and potentially penetrate the brain [239,240,241]. This is notable considering LPS is pro-epileptogenic in laboratory animals [242,243]. Therefore, the GI system should be considered as a potential therapeutic target to treat PTE.

### 7.2. Multi-Center Harmonization and Biomarker Discovery

More recently, there is a push to standardize the parameters used to study and evaluate PTE in order to increase reproducibility and translational relevance. A multicenter study demonstrated reproducible epilepsy outcomes after harmonization of key procedures, including animal housing conditions, LFPI model production, postoperative care, mortality, post-LFPI physiological monitoring, timing of blood sampling and quality, magnetic resonance imaging (MRI) timing and protocols, and duration of video-EEG follow-up [244]. This harmonization has paved the way for identification of more consistent and reliable biomarkers for PTE. In the multicenter EpiBioS4Rx cohort, acute plasma miR-212-3p levels were associated with later seizure clusters, although the tested microRNAs did not distinguish overall PTE development from its absence [245].

The identification of reliable biomarkers is essential to facilitating diagnosis and predicting long-term outcomes [246,247]. Serum-based proteins such as GFAP, S100B, neurofilament light (NF-L), and postsynaptic density protein-95 (PSD-95) have been identified as indicators of neural damage [246,248]. Following a moderate LFPI, molecular proteins show an increase in serum levels by day 3, peak at day 9, and return toward baseline by day 21 [246].

Inflammatory chemokines also serve as systemic and local biomarkers after LFPI. The chemokine CCL20 has been identified as a pro-inflammatory signal that is upregulated 24 h after LFPI in the spleen and thymus. Interestingly, CCL20 did not appear in degenerating cortical and hippocampal neurons until 48 h after injury [249]. Overall, protein, cytokine, and miRNA molecular markers reflect the presence and extent of cortical injury, pathways critical in contributing to seizure susceptibility, but have low predictive power for the development of PTE [155,245,248].

DTI is a tool for identifying microstructural white matter changes that also serve as prognostic markers for PTE. DTI metrics such as axial diffusivity, radial diffusivity, mean diffusivity, and fractional anisotropy provide detailed insights into axonal integrity and myelination. Fractional anisotropy demonstrates the highest predictive capacity 2–9 days post-injury and axial diffusivity shows the highest predictive capacity at 5 months post-injury based on axonal repair to classify TBI [247]. Damage to white matter tracts, as noted by DTI and MRI relaxation rates, have predictive power for the development of PTE. In Sprague–Dawley rats that have undergone LFPI, day 2 MRI T2 relaxation time has a 73% precision in predicting epilepsy phenotype, defined by the presence of spontaneous seizures during month-long EEG monitoring. Monitoring of pathological changes in the VPL-VPM thalamic nuclei was concluded to be involved in the development of PTE over acquiring seizure susceptibility [250]. Another study noted that LFPI induced damage to the optic tract, a thalamocortical pathway to the injured cortex, displayed axonal injury and was involved in PTE development [251].

Electrophysiological biomarkers offer further insight into circuit-level abnormalities with theta oscillations (5–12 Hz) serving as a biomarker of recovery [252]. Hippocampal and anterior cingulate cortex theta power is significantly attenuated by day 3 post-LFPI and this suppression often corresponds with acute cognitive deficits in spatial learning. The oscillations do seem to return to sham-like levels by day 7 post-injury and are associated with improved behavior performance, suggesting that neural oscillations can effectively monitor the recovery of brain function [252].

### 7.3. Advantages and Limitations of LFPI Compared to Other TBI Models

The LFPI model stands as one of the most widely used and validated preclinical platforms. Because no single experimental model perfectly encapsulates the complete clinical spectrum of TBI and subsequent epileptogenesis, understanding the specific advantages and limitations of LFPI relative to alternative models, including CCI, weight-drop, and blast-induced trauma, is essential for translational success. The primary strength of LFPI is its unique ability to mimic the mixed pathology of human TBI. Unlike the purely focal mechanical damage caused by CCI, LFPI generates a combination of focal cortical contusion and diffuse axotomy [253,254]. This closely aligns with the complex tissue damage seen in patients who go on to develop spontaneous recurrent seizures. Unlike traditional weight-drop models, which frequently fail to yield chronic epilepsy [46], LFPI has the ability to reliably produce spontaneous recurrent seizures in a relatively high percentage of injured rodents (See Table 1). LFPI also mirrors the human timeline of epileptogenesis. Rodents demonstrate a distinct, seizure-free latency period after LFPI lasting from weeks to months before developing chronic epilepsy [48]. This window is ideal for tracking emerging biomarkers and screening prophylactic anti-epileptogenic therapies. LFPI also accurately reproduces secondary hallmarks of clinical PTE. These include profound hippocampal sclerosis, physiological alterations, temporal lobe remodeling, cognitive deficits, and neuroinflammation [53].

However, there are also notable limitations of the LFPI model of PTE. To achieve a sufficient threshold of injury that triggers chronic epilepsy, researchers must use severe fluid pressure parameters. This results in a relatively high immediate mortality rate due to brainstem compression and apnea. Consistent evidence of spontaneous seizures after *moderate* FPI remains limited [58,255,256]. While LFPI simulates the intracranial pressure shifts in human trauma, the lateral pressure wave often causes an unphysiological shift in the brain tissue toward the contralateral side [28,30]. This specific biomechanical distortion differs from the direct, localized mechanical compression modeled by CCI or the linear acceleration of weight-drop systems. Moreover, emerging evidence suggests that some variations in the CCI may produce a higher incidence of PTE [257]. Therefore, in choosing PTE models, researchers should carefully consider what they are trying to model.

## 8. Conclusions

The long-term burden of TBI makes it essential to identify the mechanisms that lead from acute injury to chronic neurological disease. LFPI is a well-characterized model for investigating PTE, post-injury hyperexcitability, and associated neurobehavioral deficits. It also reproduces selected molecular and cellular changes relevant to AD and PD. Despite this experimental evidence, the contribution of individual mechanisms should not be overstated. Injury parameters and follow-up methods influence the observed outcomes, and increased seizure susceptibility should be distinguished from spontaneous epilepsy. Further studies combining standardized methods with longitudinal assessments may help identify targets for preventing or modifying the long-term consequences of TBI.

## Figures and Tables

**Figure 1:**
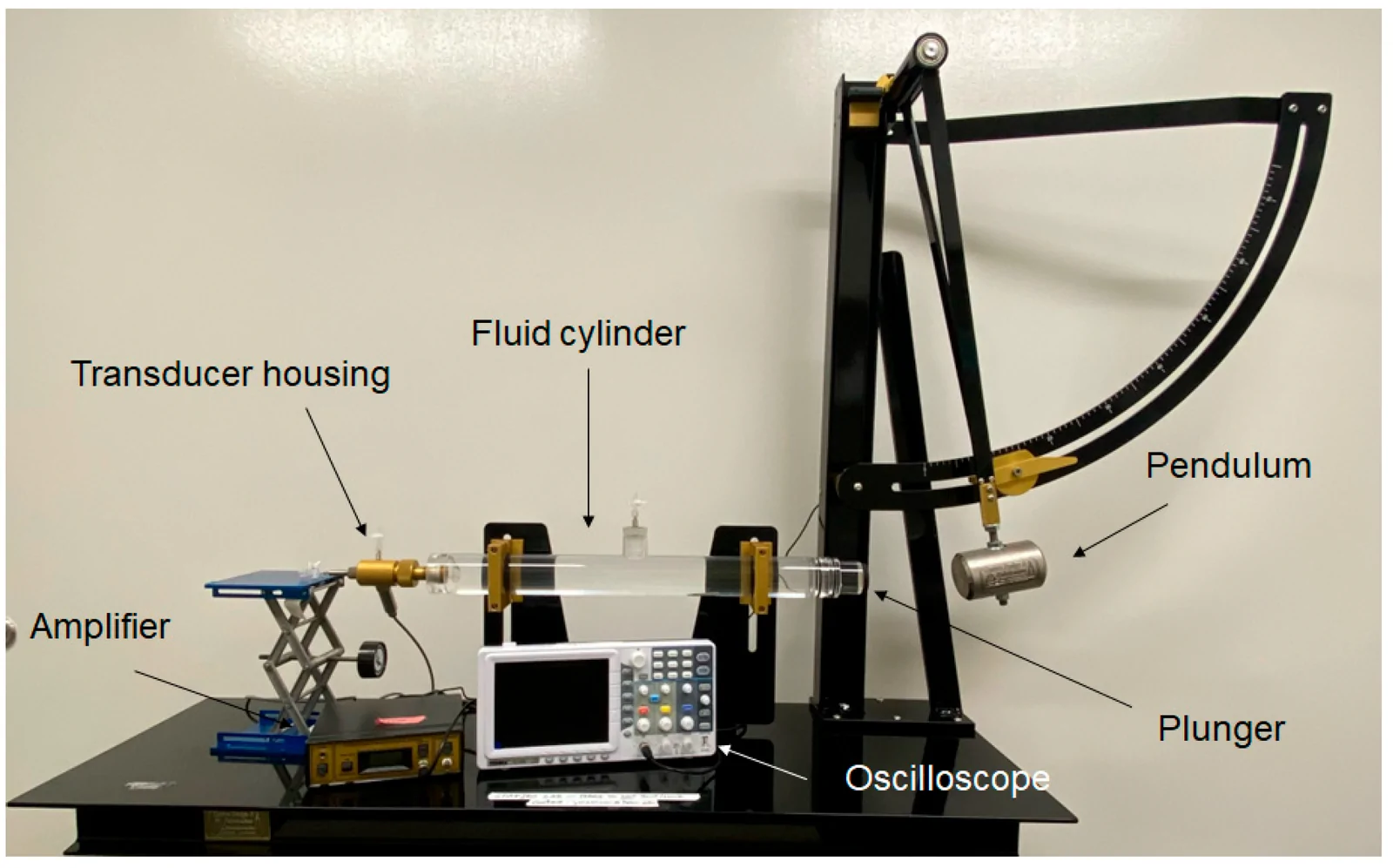
The modern fluid percussion injury device. The device shown is manufactured by Custom Design & Fabrication, Inc. (Richmond, VA, USA). It consists of a pendulum mast and a fluid cylinder, with a plunger at one end and a transducer housing at the other. An amplifier and oscilloscope measure the pressure of the fluid exiting the transducer housing.

**Table 1:** Summary of PTE study parameters in rats.

Study	Species	Strain	Sex	Coordinates (AP; ML)	Craniotomy Size	Atm	Incidence	PTE Parameter	Electrode Location	Timepoint
D’Ambrosio et al., 2004 [47]	Rat	Sprague–Dawley	M	−2.0; 3.0 (right)	3 mm	3.75–4	92%	Epileptiform activity	Perilesional/ ipsilateral	10 weeks post-LFPI
Reid et al., 2016 [50]	Rat	Sprague–Dawley	M	−3.0; 6.0 (left)	3 mm	3.2–3.5	50%	Spontaneous focal seizures	Perilesional/ ipsilateral	Week 0–52 post-LFPI
Kharatishvili et al., 2006 [51]	Rat	Sprague–Dawley	M	−2.0; 3.0 (left)	5 mm	2.6–3.3	43–50%	Spontaneous seizures	Ipsilateral and Contralateral	12–13 months post-LFPI
S. R. Shultz et al., 2013 [53]	Rat	Wistar	M	−4.0; 4.0 (right)	5 mm	3.2–3.5	52%	Spontaneous seizures; epileptic discharges	Ipsilateral and Contralateral	6 months post-LFPI
Liu et al., 2016 [54]	Rat	Wistar	M	−4.0; 4.0 (right)	5 mm	3.2–3.5	59%	Spontaneous seizures; epileptic discharges	Ipsilateral and Contralateral	12 weeks post-LFPI
Medel-Matus et al., 2021 [55]	Rat	Sprague–Dawley	M	−4.5; 3.0 (left)	5 mm	2.0	30%	Spontaneous seizures	Ipsilateral and Contralateral	7–9 months post-LFPI

## Data Availability

No new data were created or analyzed in this study. Data sharing is not applicable to this article.

## Abbreviations

The following abbreviations are used in this manuscript:

Aβ	amyloid-β
AD	Alzheimer’s disease
ADRD	Alzheimer’s disease related dementias
AMPAR	α-amino-3-hydroxy-5-methyl-4-isoxazolepropionic acid receptor
atm	atmospheres
BBB	Blood–brain barrier
BDNF	Brain-derived neurotrophic factor
CaMKII	calcium/calmodulin-dependent protein kinase II
CC	Collaborative Cross
CCI	Controlled Cortical Impact
CLIP	class II-associated invariant peptide
CNS	Central Nervous System
DAMP	Damage-associated molecular pattern
DG	Dentate Gyrus
DHRS	Dynamic high-amplitude rhythmic spikes
DTI	Diffusion Tensor Imaging
EAAT	Excitatory amino acid transporter
EEG	Electroencephalographic
EPM	Elevated plus maze
ER	Endoplasmic reticulum
ETC	Electron transport chain
FPI	Fluid Percussion Injury
FST	Forced Swim Test
GCS	Glasgow Coma Scale
HMGB1	High mobility group box 1
IED	Interictal epileptiform discharges
IEG	Immediate early gene
IL	interleukin
KA	Kainic acid
LFPI	Lateral Fluid Percussion Injury
LPS	lipopolysaccharide
MDD	Major Depressive Disorder
MIF	Macrophage migration inhibitory factor
MRI	Magnetic Resonance Imaging
MTLE	Mesial temporal lobe epilepsy
MWM	Morris water maze
NF-L	Neurofilament light
NMDAR	N-methyl-D-aspartate receptor
NSS	Neurological Severity Score
PAL	Pair associate learning
PD	Parkinson’s disease
PSD	Post-synaptic density
p-tau	Phosphorylated tau
PTE	Post-Traumatic Epilepsy
PTZ	pentylenetetrazol
PV-INs	Parvalbumin-expressing interneurons
ROS	Reactive oxygen species
TBI	Traumatic Brain Injury
TLR	Toll-like receptor
TNF-α	Tumor necrosis factor-α
TREM2	triggering receptor expressed on myeloid cells 2
UPR	Unfolded protein response
vWF	von Willebrand factor

## References

[1] Corso P., Finkelstein E., Miller T., Fiebelkorn I., Zaloshnja E. (2006). Incidence and lifetime costs of injuries in the United States. Inj. Prev..

[2] Taylor C.A., Bell J.M., Breiding M.J., Xu L. (2017). Traumatic Brain Injury-Related Emergency Department Visits, Hospitalizations, and Deaths—United States, 2007 and 2013. MMWR Surveill. Summ..

[3] Schneider A.L.C., Wang D., Ling G., Gottesman R.F., Selvin E. (2018). Prevalence of Self-Reported Head Injury in the United States. N. Engl. J. Med..

[4] Mukherjee S., Arisi G.M., Mims K., Hollingsworth G., O’Neil K., Shapiro L.A. (2020). Neuroinflammatory mechanisms of post-traumatic epilepsy. J. Neuroinflamm..

[5] Li Y., Li Y., Li X., Zhang S., Zhao J., Zhu X., Tian G. (2017). Head Injury as a Risk Factor for Dementia and Alzheimer’s Disease: A Systematic Review and Meta-Analysis of 32 Observational Studies. PLoS ONE.

[6] Gardner R.C., Byers A.L., Barnes D.E., Li Y., Boscardin J., Yaffe K. (2018). Mild TBI and risk of Parkinson disease: A Chronic Effects of Neurotrauma Consortium Study. Neurology.

[7] Jafari S., Etminan M., Aminzadeh F., Samii A. (2013). Head injury and risk of Parkinson disease: A systematic review and meta-analysis. Mov. Disord..

[8] Perry D.C., Sturm V.E., Peterson M.J., Pieper C.F., Bullock T., Boeve B.F., Miller B.L., Guskiewicz K.M., Berger M.S., Kramer J.H. (2016). Association of traumatic brain injury with subsequent neurological and psychiatric disease: A meta-analysis. J. Neurosurg..

[9] Pease M., Mallela A.N., Elmer J., Okonkwo D.O., Shutter L., Barot N., Gonzalez-Martinez J., Castellano J.F. (2023). Association of Posttraumatic Epilepsy With Long-term Functional Outcomes in Individuals With Severe Traumatic Brain Injury. Neurology.

[10] Orman J.A., Geyer D., Jones J., Schneider E.B., Grafman J., Pugh M.J., Dubose J. (2012). Epidemiology of moderate-to-severe penetrating versus closed traumatic brain injury in the Iraq and Afghanistan wars. J. Trauma Acute Care Surg..

[11] Mattei T.A., Teasdale G.M. (2020). The Story of the Development and Adoption of the Glasgow Coma Scale: Part I, The Early Years. World Neurosurg..

[12] Teasdale G., Jennett B. (1974). Assessment of Coma and Impaired Consciousness: A Practical Scale. Lancet.

[13] Duret H. (1878). Etudes Expérimentales sur les Traumatismes Cérébraux. Imprimerie et Stéréotypie Cerf et Fils. Ph.D. Thesis.

[14] Ward A.A. (1958). Physiological Basis of Concussion. J. Neurosurg..

[15] Foltz E.L., Jenkner F.L., Ward A.A. (1953). Experimental Cerebral Concussion. J. Neurosurg..

[16] Gurdjian E.S., Lissner H.R., Webster J.E., Latimer F.R., Haddad B.F. (1954). Studies on experimental concussion: Relation of physiologic effect to time duration of intracranial pressure increase at impact. Neurology.

[17] Walker A.E., Kollros J.J., Case T.J. (1944). The Physiological Basis of Concussion. J. Neurosurg..

[18] Govons S.R., Govons R.B., VanHuss W.D., Heusner W.W. (1972). Brain concussion in the rat. Exp. Neurol..

[19] Denny-Brown D.E., Russell W.R. (1941). Experimental Concussion: (Section of Neurology). Proc. R. Soc. Med..

[20] Stitt D., Kabaliuk N., Alexander K., Draper N. (2024). Potential of Soft-Shelled Rugby Headgear to Lower Regional Brain Strain Metrics During Standard Drop Tests. Sports Med. Open.

[21] Alder J., Fujioka W., Lifshitz J., Crockett D.P., Thakker-Varia S. (2011). Lateral fluid percussion: Model of traumatic brain injury in mice. J. Vis. Exp..

[22] Lindgren S., Rinder L. (1966). Experimental studies in head injury. II. Pressure propagation in “percussion concussion”. Biophysik.

[23] Lindgren S., Rinder L. (1969). Production and distribution of intracranial and intraspinal pressure changes at sudden extradural fluid volume input in rabbits. Acta Physiol. Scand..

[24] Rinder L. (1969). “Concussive response” and intracranial pressure changes at sudden extradural fluid volume input in rabbits. Acta Physiol. Scand..

[25] Sullivan H.G., Martinez J., Becker D.P., Miller J.D., Griffith R., Wist A.O. (1976). Fluid-percussion model of mechanical brain injury in the cat. J. Neurosurg..

[26] Dixon C.E., Lyeth B.G., Povlishock J.T., Findling R.L., Hamm R.J., Marmarou A., Young H.F., Hayes R.L. (1987). A fluid percussion model of experimental brain injury in the rat. J. Neurosurg..

[27] Lyeth B.G., Dixon C.E., Jenkins L.W., Hamm R.J., Alberico A., Young H.F., Stonnington H.H., Hayes R.L. (1988). Effects of scopolamine treatment on long-term behavioral deficits following concussive brain injury to the rat. Brain Res..

[28] Witcher K.G., Dziabis J.E., Bray C.E., Gordillo A.J., Kumar J.E., Eiferman D.S., Godbout J.P., Kokiko-Cochran O.N. (2020). Comparison between midline and lateral fluid percussion injury in mice reveals prolonged but divergent cortical neuroinflammation. Brain Res..

[29] Grady M.S., Charleston J.S., Maris D., Witgen B.M., Lifshitz J. (2003). Neuronal and glial cell number in the hippocampus after experimental traumatic brain injury: Analysis by stereological estimation. J. Neurotrauma.

[30] Cortez S.C., McIntosh T.K., Noble L.J. (1989). Experimental fluid percussion brain injury: Vascular disruption and neuronal and glial alterations. Brain Res..

[31] McIntosh T.K., Vink R., Noble L., Yamakami I., Fernyak S., Soares H., Faden A.L. (1989). Traumatic brain injury in the rat: Characterization of a lateral fluid-percussion model. Neuroscience.

[32] Lowenstein D.H., Thomas M.J., Smith D.H., McIntosh T.K. (1992). Selective vulnerability of dentate hilar neurons following traumatic brain injury: A potential mechanistic link between head trauma and disorders of the hippocampus. J. Neurosci..

[33] Toth Z., Hollrigel G.S., Gorcs T., Soltesz I. (1997). Instantaneous perturbation of dentate interneuronal networks by a pressure wave-transient delivered to the neocortex. J. Neurosci..

[34] Carbonell W.S., Maris D.O., McCall T., Grady M.S. (1998). Adaptation of the fluid percussion injury model to the mouse. J. Neurotrauma.

[35] Laskowitz D., Grant G. (2016). Frontiers in Neuroscience. Translational Research in Traumatic Brain Injury.

[36] Kobau R., Luncheon C., Greenlund K. (2023). Active epilepsy prevalence among U.S. adults is 1.1% and differs by educational level-National Health Interview Survey, United States, 2021. Epilepsy Behav..

[37] Nguyen R., Téllez Zenteno J.F. (2009). Injuries in epilepsy: A review of its prevalence, risk factors, type of injuries and prevention. Neurol. Int..

[38] Verellen R.M., Cavazos J.E. (2010). Post-traumatic epilepsy: An overview. Therapy.

[39] Frey L.C. (2003). Epidemiology of posttraumatic epilepsy: A critical review. Epilepsia.

[40] Salazar A.M., Jabbari B., Vance S.C., Grafman J., Amin D., Dillon J.D. (1985). Epilepsy after penetrating head injury. I. Clinical correlates: A report of the Vietnam Head Injury Study. Neurology.

[41] Awan N., Kumar R.G., Juengst S.B., DiSanto D., Harrison-Felix C., Dams-O’Connor K., Pugh M.J., Zafonte R.D., Walker W.C., Szaflarski J.P. (2025). Development of individualized risk assessment models for predicting post-traumatic epilepsy 1 and 2 years after moderate-to-severe traumatic brain injury: A traumatic brain injury model system study. Epilepsia.

[42] Acosta O., Barcikowski J. (2024). An Update on Post-Traumatic Epilepsy. Curr. Phys. Med. Rehabil. Rep..

[43] Irimia A., Van Horn J.D. (2015). Epileptogenic focus localization in treatment-resistant post-traumatic epilepsy. J. Clin. Neurosci..

[44] Larkin M., Meyer R.M., Szuflita N.S., Severson M.A., Levine Z.T. (2016). Post-Traumatic, Drug-Resistant Epilepsy and Review of Seizure Control Outcomes from Blinded, Randomized Controlled Trials of Brain Stimulation Treatments for Drug-Resistant Epilepsy. Cureus.

[45] Gugger J.J., Kennedy E., Panahi S., Tate D.F., Roghani A., Van Cott A.C., Lopez M.R., Altalib H., Diaz-Arrastia R., Pugh M.J. (2022). Multimodal Quality of Life Assessment in Post-9/11 Veterans With Epilepsy: Impact of Drug Resistance, Traumatic Brain Injury, and Comorbidity. Neurology.

[46] Brady R.D., Casillas-Espinosa P.M., Agoston D.V., Bertram E.H., Kamnaksh A., Semple B.D., Shultz S.R. (2019). Modelling traumatic brain injury and posttraumatic epilepsy in rodents. Neurobiol. Dis..

[47] D’Ambrosio R., Fairbanks J.P., Fender J.S., Born D.E., Doyle D.L., Miller J.W. (2004). Post-traumatic epilepsy following fluid percussion injury in the rat. Brain.

[48] D’Ambrosio R., Fender J.S., Fairbanks J.P., Simon E.A., Born D.E., Doyle D.L., Miller J.W. (2005). Progression from frontal-parietal to mesial-temporal epilepsy after fluid percussion injury in the rat. Brain.

[49] D’Ambrosio R., Hakimian S., Stewart T., Verley D.R., Fender J.S., Eastman C.L., Sheerin A.H., Gupta P., Diaz-Arrastia R., Ojemann J. (2009). Functional definition of seizure provides new insight into post-traumatic epileptogenesis. Brain.

[50] Reid A.Y., Bragin A., Giza C.C., Staba R.J., Engel J. (2016). The progression of electrophysiologic abnormalities during epileptogenesis after experimental traumatic brain injury. Epilepsia.

[51] Kharatishvili I., Nissinen J.P., McIntosh T.K., Pitkanen A. (2006). A model of posttraumatic epilepsy induced by lateral fluid-percussion brain injury in rats. Neuroscience.

[52] Kharatishvili I., Immonen R., Gröhn O., Pitkänen A. (2007). Quantitative diffusion MRI of hippocampus as a surrogate marker for post-traumatic epileptogenesis. Brain.

[53] Shultz S.R., Cardamone L., Liu Y.R., Hogan R.E., Maccotta L., Wright D.K., Zheng P., Koe A., Gregoire M.C., Williams J.P. (2013). Can structural or functional changes following traumatic brain injury in the rat predict epileptic outcome?. Epilepsia.

[54] Liu S.J., Zheng P., Wright D.K., Dezsi G., Braine E., Nguyen T., Corcoran N.M., Johnston L.A., Hovens C.M., Mayo J.N. (2016). Sodium selenate retards epileptogenesis in acquired epilepsy models reversing changes in protein phosphatase 2A and hyperphosphorylated tau. Brain.

[55] Medel-Matus J.S., Shin D., Sankar R., Mazarati A. (2021). Diversity of kindling of limbic seizures after lateral fluid percussion injury in the rat. Epilepsia Open.

[56] Smith D., Rau T., Poulsen A., MacWilliams Z., Patterson D., Kelly W., Poulsen D. (2018). Convulsive seizures and EEG spikes after lateral fluid-percussion injury in the rat. Epilepsy Res..

[57] Lapinlampi N., Andrade P., Paananen T., Hämäläinen E., Ekolle Ndode-Ekane X., Puhakka N., Pitkänen A. (2020). Postinjury weight rather than cognitive or behavioral impairment predicts development of posttraumatic epilepsy after lateral fluid-percussion injury in rats. Epilepsia.

[58] Kharatishvili I., Pitkänen A. (2010). Association of the severity of cortical damage with the occurrence of spontaneous seizures and hyperexcitability in an animal model of posttraumatic epilepsy. Epilepsy Res..

[59] Leung W.L., Dill L.K., Perucca P., O’Brien T.J., Casillas-Espinosa P.M., Semple B.D. (2023). Inherent Susceptibility to Acquired Epilepsy in Selectively Bred Rats Influences the Acute Response to Traumatic Brain Injury. J. Neurotrauma.

[60] Liu Y., Liu X., Chen Z., Wang Y., Li J., Gong J., He A., Zhao M., Yang C., Yang W. (2022). Evaluation of decompressive craniectomy in mice after severe traumatic brain injury. Front. Neurol..

[61] Lifshitz J., Rowe R.K., Griffiths D.R., Evilsizor M.N., Thomas T.C., Adelson P.D., McIntosh T.K. (2016). Clinical relevance of midline fluid percussion brain injury: Acute deficits, chronic morbidities and the utility of biomarkers. Brain Inj..

[62] Bolkvadze T., Pitkanen A. (2012). Development of post-traumatic epilepsy after controlled cortical impact and lateral fluid-percussion-induced brain injury in the mouse. J. Neurotrauma.

[63] Shannon T., Cotter C., Fitzgerald J., Houle S., Levine N., Shen Y., Rajjoub N., Dobres S., Iyer S., Xenakis J. (2024). Genetic diversity drives extreme responses to traumatic brain injury and post-traumatic epilepsy. Exp. Neurol..

[64] Shannon T., Levine N., Dirickson R., Shen Y., Cotter C., Rajjoub N., Fitzgerald J., Pardo-Manuel de Villena F., Kokiko-Cochran O., Gu B. (2024). Early hippocampal high-amplitude rhythmic spikes predict post-traumatic epilepsy in mice. Front. Neurosci..

[65] Pitkänen A., Immonen R. (2014). Epilepsy related to traumatic brain injury. Neurotherapeutics.

[66] Mukherjee S., Zeitouni S., Cavarsan C.F., Shapiro L.A. (2013). Increased seizure susceptibility in mice 30 days after fluid percussion injury. Front. Neurol..

[67] Andrade P., Lara-Valderrábano L., Manninen E., Ciszek R., Tapiala J., Ndode-Ekane X.E., Pitkänen A. (2022). Seizure Susceptibility and Sleep Disturbance as Biomarkers of Epileptogenesis after Experimental TBI. Biomedicines.

[68] Huusko N., Pitkänen A. (2014). Parvalbumin immunoreactivity and expression of GABAA receptor subunits in the thalamus after experimental TBI. Neuroscience.

[69] Mishra A.M., Bai X., Sanganahalli B.G., Waxman S.G., Shatillo O., Grohn O., Hyder F., Pitkänen A., Blumenfeld H. (2014). Decreased resting functional connectivity after traumatic brain injury in the rat. PLoS ONE.

[70] Silva L.F., Hoffmann M.S., Rambo L.M., Ribeiro L.R., Lima F.D., Furian A.F., Oliveira M.S., Fighera M.R., Royes L.F. (2011). The involvement of Na+, K+-ATPase activity and free radical generation in the susceptibility to pentylenetetrazol-induced seizures after experimental traumatic brain injury. J. Neurol. Sci..

[71] Zanier E.R., Lee S.M., Vespa P.M., Giza C.C., Hovda D.A. (2003). Increased hippocampal CA3 vulnerability to low-level kainic acid following lateral fluid percussion injury. J. Neurotrauma.

[72] Mazarati A., Medel-Matus J.S., Shin D., Jacobs J.P., Sankar R. (2021). Disruption of intestinal barrier and endotoxemia after traumatic brain injury: Implications for post-traumatic epilepsy. Epilepsia.

[73] Wang Y., Andrade P., Pitkänen A. (2021). Peripheral Infection after Traumatic Brain Injury Augments Excitability in the Perilesional Cortex and Dentate Gyrus. Biomedicines.

[74] Folweiler K.A., Samuel S., Metheny H.E., Cohen A.S. (2018). Diminished Dentate Gyrus Filtering of Cortical Input Leads to Enhanced Area Ca3 Excitability after Mild Traumatic Brain Injury. J. Neurotrauma.

[75] Folweiler K.A., Xiong G., Best K.M., Metheny H.E., Nah G., Cohen A.S. (2020). Traumatic Brain Injury Diminishes Feedforward Activation of Parvalbumin-Expressing Interneurons in the Dentate Gyrus. eNeuro.

[76] Witgen B.M., Lifshitz J., Smith M.L., Schwarzbach E., Liang S.L., Grady M.S., Cohen A.S. (2005). Regional hippocampal alteration associated with cognitive deficit following experimental brain injury: A systems, network and cellular evaluation. Neuroscience.

[77] Santhakumar V., Bender R., Frotscher M., Ross S.T., Hollrigel G.S., Toth Z., Soltesz I. (2000). Granule cell hyperexcitability in the early post-traumatic rat dentate gyrus: The ‘irritable mossy cell’ hypothesis. J. Physiol..

[78] Santhakumar V., Ratzliff A.D., Jeng J., Toth Z., Soltesz I. (2001). Long-term hyperexcitability in the hippocampus after experimental head trauma. Ann. Neurol..

[79] Gupta A., Elgammal F.S., Proddutur A., Shah S., Santhakumar V. (2012). Decrease in tonic inhibition contributes to increase in dentate semilunar granule cell excitability after brain injury. J. Neurosci..

[80] Tran L.D., Lifshitz J., Witgen B.M., Schwarzbach E., Cohen A.S., Grady M.S. (2006). Response of the contralateral hippocampus to lateral fluid percussion brain injury. J. Neurotrauma.

[81] Gupta A., Dovek L., Proddutur A., Elgammal F.S., Santhakumar V. (2022). Long-Term Effects of Moderate Concussive Brain Injury During Adolescence on Synaptic and Tonic GABA Currents in Dentate Granule Cells and Semilunar Granule Cells. Front. Neurosci..

[82] Corrubia L., Huang A., Nguyen S., Shiflett M.W., Jones M.V., Ewell L.A., Santhakumar V. (2023). Early deficits in dentate circuit and behavioral pattern separation after concussive brain injury. Exp. Neurol..

[83] Hsieh T.H., Lee H.H.C., Hameed M.Q., Pascual-Leone A., Hensch T.K., Rotenberg A. (2017). Trajectory of Parvalbumin Cell Impairment and Loss of Cortical Inhibition in Traumatic Brain Injury. Cereb. Cortex.

[84] Akasu T., Muraoka N., Hasuo H. (2002). Hyperexcitability of hippocampal CA1 neurons after fluid percussion injury of the rat cerebral cortex. Neurosci. Lett..

[85] Koch P.F., Cottone C., Adam C.D., Ulyanova A.V., Russo R.J., Weber M.T., Arena J.D., Johnson V.E., Wolf J.A. (2020). Traumatic Brain Injury Preserves Firing Rates But Disrupts Laminar Oscillatory Coupling and Neuronal Entrainment in Hippocampal CA1. eNeuro.

[86] Chirumamilla S., Sun D., Bullock M.R., Colello R.J. (2002). Traumatic brain injury induced cell proliferation in the adult mammalian central nervous system. J. Neurotrauma.

[87] Shapiro L.A. (2017). Altered Hippocampal Neurogenesis during the First 7 Days after a Fluid Percussion Traumatic Brain Injury. Cell Transplant..

[88] Correll E.A., Ramser B.J., Knott M.V., McCullumsmith R.E., McGuire J.L., Ngwenya L.B. (2021). Deficits in pattern separation and dentate gyrus proliferation after rodent lateral fluid percussion injury. IBRO Neurosci. Rep..

[89] Clark L.R., Yun S., Acquah N.K., Kumar P.L., Metheny H.E., Paixao R.C.C., Cohen A.S., Eisch A.J. (2020). Mild Traumatic Brain Injury Induces Transient, Sequential Increases in Proliferation, Neuroblasts/Immature Neurons, and Cell Survival: A Time Course Study in the Male Mouse Dentate Gyrus. Front. Neurosci..

[90] Iannucci J., John M., Oderinde S., Arisi G.M., Arismendi V., Pereverzev A., Shapiro L.A. (2026). CD44 antagonism after traumatic brain injury influences the adult neurogenic niche and behavioral outcomes. Brain Struct. Funct..

[91] Iannucci J., Venkatasamy L., Davis M., Nguyen T.A., Yadav G.S., Nugeness V., Dominy R., Arisi G.M., Newell-Rogers M.K., Shapiro L.A. (2026). Sex differences in neurobehavior and the adult hippocampal neurogenic niche: Influence of traumatic brain injury and CLIP antagonism. Front. Behav. Neurosci..

[92] Robinson C., Apgar C., Shapiro L.A. (2016). Astrocyte Hypertrophy Contributes to Aberrant Neurogenesis after Traumatic Brain Injury. Neural Plast..

[93] Neuberger E.J., Swietek B., Corrubia L., Prasanna A., Santhakumar V. (2017). Enhanced Dentate Neurogenesis after Brain Injury Undermines Long-Term Neurogenic Potential and Promotes Seizure Susceptibility. Stem Cell Rep..

[94] Cho K.O., Lybrand Z.R., Ito N., Brulet R., Tafacory F., Zhang L., Good L., Ure K., Kernie S.G., Birnbaum S.G. (2015). Aberrant hippocampal neurogenesis contributes to epilepsy and associated cognitive decline. Nat. Commun..

[95] Shapiro L.A., Ribak C.E. (2006). Newly born dentate granule neurons after pilocarpine-induced epilepsy have hilar basal dendrites with immature synapses. Epilepsy Res..

[96] Shapiro L.A., Figueroa-Aragon S., Ribak C.E. (2007). Newly generated granule cells show rapid neuroplastic changes in the adult rat dentate gyrus during the first five days following pilocarpine-induced seizures. Eur. J. Neurosci..

[97] Shapiro L.A., Wang L., Upadhyaya P., Ribak C.E. (2011). Seizure-induced Increased Neurogenesis Occurs in the Dentate Gyrus of Aged Sprague-Dawley Rats. Aging Dis..

[98] Iyengar S.S., LaFrancois J.J., Friedman D., Drew L.J., Denny C.A., Burghardt N.S., Wu M.V., Hsieh J., Hen R., Scharfman H.E. (2015). Suppression of adult neurogenesis increases the acute effects of kainic acid. Exp. Neurol..

[99] Overstreet-Wadiche L.S., Bromberg D.A., Bensen A.L., Westbrook G.L. (2006). Seizures accelerate functional integration of adult-generated granule cells. J. Neurosci..

[100] Parent J.M., Lowenstein D.H. (2002). Seizure-induced neurogenesis: Are more new neurons good for an adult brain?. Prog. Brain Res..

[101] Walter C., Murphy B.L., Pun R.Y., Spieles-Engemann A.L., Danzer S.C. (2007). Pilocarpine-induced seizures cause selective time-dependent changes to adult-generated hippocampal dentate granule cells. J. Neurosci..

[102] Jessberger S., Zhao C., Toni N., Clemenson G.D., Li Y., Gage F.H. (2007). Seizure-associated, aberrant neurogenesis in adult rats characterized with retrovirus-mediated cell labeling. J. Neurosci..

[103] Shapiro L.A., Korn M.J., Shan Z., Ribak C.E. (2005). GFAP-expressing radial glia-like cell bodies are involved in a one-to-one relationship with doublecortin-immunolabeled newborn neurons in the adult dentate gyrus. Brain Res..

[104] Scharfman H.E., Goodman J.H., Sollas A.L. (2000). Granule-like neurons at the hilar/CA3 border after status epilepticus and their synchrony with area CA3 pyramidal cells: Functional implications of seizure-induced neurogenesis. J. Neurosci..

[105] Scharfman H.E. (2004). Functional implications of seizure-induced neurogenesis. Adv. Exp. Med. Biol..

[106] Scharfman H.E., Sollas A.E., Berger R.E., Goodman J.H., Pierce J.P. (2003). Perforant path activation of ectopic granule cells that are born after pilocarpine-induced seizures. Neuroscience.

[107] McCloskey D.P., Hintz T.M., Pierce J.P., Scharfman H.E. (2006). Stereological methods reveal the robust size and stability of ectopic hilar granule cells after pilocarpine-induced status epilepticus in the adult rat. Eur. J. Neurosci..

[108] Pierce J.P., Melton J., Punsoni M., McCloskey D.P., Scharfman H.E. (2005). Mossy fibers are the primary source of afferent input to ectopic granule cells that are born after pilocarpine-induced seizures. Exp. Neurol..

[109] Pierce J.P., Punsoni M., McCloskey D.P., Scharfman H.E. (2007). Mossy cell axon synaptic contacts on ectopic granule cells that are born following pilocarpine-induced seizures. Neurosci. Lett..

[110] Chen L., Xu Y., Cheng H., Li Z., Lai N., Li M., Ruan Y., Zheng Y., Fei F., Xu C. (2023). Adult-born neurons in critical period maintain hippocampal seizures via local aberrant excitatory circuits. Signal Transduct. Target. Ther..

[111] Morgan R.J., Soltesz I. (2008). Nonrandom connectivity of the epileptic dentate gyrus predicts a major role for neuronal hubs in seizures. Proc. Natl. Acad. Sci. USA.

[112] Jessberger S., Gage F.H. (2014). Adult neurogenesis: Bridging the gap between mice and humans. Trends Cell Biol..

[113] Dumitru I., Paterlini M., Zamboni M., Ziegenhain C., Giatrellis S., Saghaleyni R., Björklund Å., Alkass K., Tata M., Druid H. (2025). Identification of proliferating neural progenitors in the adult human hippocampus. Science.

[114] Duque A., Arellano J.I., Rakic P. (2022). An assessment of the existence of adult neurogenesis in humans and value of its rodent models for neuropsychiatric diseases. Mol. Psychiatry.

[115] Loup F., Wieser H.G., Yonekawa Y., Aguzzi A., Fritschy J.M. (2000). Selective alterations in GABAA receptor subtypes in human temporal lobe epilepsy. J. Neurosci..

[116] Drexel M., Puhakka N., Kirchmair E., Hörtnagl H., Pitkänen A., Sperk G. (2015). Expression of GABA receptor subunits in the hippocampus and thalamus after experimental traumatic brain injury. Neuropharmacology.

[117] Hameed M.Q., Hsieh T.H., Morales-Quezada L., Lee H.H.C., Damar U., MacMullin P.C., Hensch T.K., Rotenberg A. (2019). Ceftriaxone Treatment Preserves Cortical Inhibitory Interneuron Function via Transient Salvage of GLT-1 in a Rat Traumatic Brain Injury Model. Cereb. Cortex.

[118] Therajaran P., Hamilton J.A., O’Brien T.J., Jones N.C., Ali I. (2020). Microglial polarization in posttraumatic epilepsy: Potential mechanism and treatment opportunity. Epilepsia.

[119] Newell-Rogers M.K., Rogers S.K., Tobin R.P., Mukherjee S., Shapiro L.A. (2020). Antagonism of Macrophage Migration Inhibitory Factory (MIF) after Traumatic Brain Injury Ameliorates Astrocytosis and Peripheral Lymphocyte Activation and Expansion. Int. J. Mol. Sci..

[120] Bedner P., Dupper A., Hüttmann K., Müller J., Herde M.K., Dublin P., Deshpande T., Schramm J., Häussler U., Haas C.A. (2015). Astrocyte uncoupling as a cause of human temporal lobe epilepsy. Brain.

[121] Devinsky O., Vezzani A., Najjar S., De Lanerolle N.C., Rogawski M.A. (2013). Glia and epilepsy: Excitability and inflammation. Trends Neurosci..

[122] Vezzani A., Ravizza T., Bedner P., Aronica E., Steinhauser C., Boison D. (2022). Astrocytes in the initiation and progression of epilepsy. Nat. Rev. Neurol..

[123] Dorsett C.R., McGuire J.L., Niedzielko T.L., DePasquale E.A., Meller J., Floyd C.L., McCullumsmith R.E. (2017). Traumatic Brain Injury Induces Alterations in Cortical Glutamate Uptake without a Reduction in Glutamate Transporter-1 Protein Expression. J. Neurotrauma.

[124] Gao Y., Liu N., Chen J., Zheng P., Niu J., Tang S., Peng X., Wu J., Yu J., Ma L. (2023). Neuropharmacological insight into preventive intervention in posttraumatic epilepsy based on regulating glutamate homeostasis. CNS Neurosci. Ther..

[125] Dorsett C.R., McGuire J.L., DePasquale E.A.K., Gardner A.E., Floyd C.L., McCullumsmith R.E. (2016). Glutamate Neurotransmission in Rodent Models of Traumatic Brain Injury. J. Neurotrauma.

[126] Willmore L.J., Ueda Y. (2009). Posttraumatic epilepsy: Hemorrhage, free radicals and the molecular regulation of glutamate. Neurochem. Res..

[127] Komoltsev I.G., Tret’yakova L.V., Frankevich S.O., Shirobokova N.I., Volkova A.A., Butuzov A.V., Novikova M.R., Kvichansky A.A., Moiseeva Y.V., Onufriev M.V. (2022). Neuroinflammatory Cytokine Response, Neuronal Death, and Microglial Proliferation in the Hippocampus of Rats During the Early Period After Lateral Fluid Percussion-Induced Traumatic Injury of the Neocortex. Mol. Neurobiol..

[128] Korgaonkar A.A., Li Y., Sekhar D., Subramanian D., Guevarra J., Swietek B., Pallottie A., Singh S., Kella K., Elkabes S. (2020). Toll-like Receptor 4 Signaling in Neurons Enhances Calcium-Permeable alpha-Amino-3-Hydroxy-5-Methyl-4-Isoxazolepropionic Acid Receptor Currents and Drives Post-Traumatic Epileptogenesis. Ann. Neurol..

[129] Wang Y., Hameed M.Q., Rakhade S.N., Iglesias A.H., Muller P.A., Mou D.L., Rotenberg A. (2014). Hippocampal immediate early gene transcription in the rat fluid percussion traumatic brain injury model. Neuroreport.

[130] Austin J.E., Buckmaster P.S. (2004). Recurrent excitation of granule cells with basal dendrites and low interneuron density and inhibitory postsynaptic current frequency in the dentate gyrus of macaque monkeys. J. Comp. Neurol..

[131] Seress L., Mrzljak L. (1987). Basal dendrites of granule cells are normal features of the fetal and adult dentate gyrus of both monkey and human hippocampal formations. Brain Res..

[132] Yan X.X., Spigelman I., Tran P.H., Ribak C.E. (2001). Atypical features of rat dentate granule cells: Recurrent basal dendrites and apical axons. Anat. Embryol..

[133] Bray C.E., Witcher K.G., Adekunle-Adegbite D., Ouvina M., Witzel M., Hans E., Tapp Z.M., Packer J., Goodman E., Zhao F. (2022). Chronic Cortical Inflammation, Cognitive Impairment, and Immune Reactivity Associated with Diffuse Brain Injury Are Ameliorated by Forced Turnover of Microglia. J. Neurosci..

[134] Norden D.M., Muccigrosso M.M., Godbout J.P. (2015). Microglial priming and enhanced reactivity to secondary insult in aging, and traumatic CNS injury, and neurodegenerative disease. Neuropharmacology.

[135] Witcher K.G., Bray C.E., Dziabis J.E., McKim D.B., Benner B.N., Rowe R.K., Kokiko-Cochran O.N., Popovich P.G., Lifshitz J., Eiferman D.S. (2018). Traumatic brain injury-induced neuronal damage in the somatosensory cortex causes formation of rod-shaped microglia that promote astrogliosis and persistent neuroinflammation. Glia.

[136] Sharma S., Tiarks G., Haight J., Bassuk A.G. (2021). Neuropathophysiological Mechanisms and Treatment Strategies for Post-traumatic Epilepsy. Front. Mol. Neurosci..

[137] Sun L., Shan W., Yang H., Liu R., Wu J., Wang Q. (2021). The Role of Neuroinflammation in Post-traumatic Epilepsy. Front. Neurol..

[138] Komoltsev I.G., Sinkin M.V., Volkova A.A., Smirnova E.A., Novikova M.R., Kordonskaya O.O., Talypov A.E., Guekht A.B., Krylov V.V., Gulyaeva N.V. (2020). A Translational Study on Acute Traumatic Brain Injury: High Incidence of Epileptiform Activity on Human and Rat Electrocorticograms and Histological Correlates in Rats. Brain Sci..

[139] Ekdahl C.T., Claasen J.H., Bonde S., Kokaia Z., Lindvall O. (2003). Inflammation is detrimental for neurogenesis in adult brain. Proc. Natl. Acad. Sci. USA.

[140] Houle S., Tapp Z., Dobres S., Ahsan S., Reyes Y., Cotter C., Mitsch J., Zimomra Z., Peng J., Rowe R.K. (2024). Sleep fragmentation after traumatic brain injury impairs behavior and conveys long-lasting impacts on neuroinflammation. Brain Behav. Immun. Health.

[141] Molcanyi M., Riess P., Bentz K., Maegele M., Hescheler J., Schäfke B., Trapp T., Neugebauer E., Klug N., Schäfer U. (2007). Trauma-associated inflammatory response impairs embryonic stem cell survival and integration after implantation into injured rat brain. J. Neurotrauma.

[142] Andoh M., Ikegaya Y., Koyama R. (2019). Synaptic Pruning by Microglia in Epilepsy. J. Clin. Med..

[143] DeSana A.J., Alfawares Y., Khatri R., Hopkins T.M., Best F.V., McGuire J.L., Ngwenya L.B. (2025). Altered Neuroinflammatory Transcriptomic Profile in the Hippocampal Dentate Gyrus Three Weeks After Lateral Fluid Percussion Injury in Rats. Int. J. Mol. Sci..

[144] Villasana-Salazar B., Vezzani A. (2023). Neuroinflammation microenvironment sharpens seizure circuit. Neurobiol. Dis..

[145] Vezzani A., Balosso S., Ravizza T. (2019). Neuroinflammatory pathways as treatment targets and biomarkers in epilepsy. Nat. Rev. Neurol..

[146] Tan H.P., Guo Q., Hua G., Chen J.X., Liang J.C. (2018). Inhibition of endoplasmic reticulum stress alleviates secondary injury after traumatic brain injury. Neural Regen. Res..

[147] Folbergrová J., Kunz W.S. (2012). Mitochondrial dysfunction in epilepsy. Mitochondrion.

[148] Jiang X.B., Kuroiwa T., Ohno K., Duan L., Aoyagi M., Hirakawa K. (1999). Local mitochondrial function following traumatic brain injury in rats. Neurol. Med. Chir..

[149] Xing G., Barry E.S., Benford B., Grunberg N.E., Li H., Watson W.D., Sharma P. (2013). Impact of repeated stress on traumatic brain injury-induced mitochondrial electron transport chain expression and behavioral responses in rats. Front. Neurol..

[150] Lifshitz J., Friberg H., Neumar R.W., Raghupathi R., Welsh F.A., Janmey P., Saatman K.E., Wieloch T., Grady M.S., McIntosh T.K. (2003). Structural and functional damage sustained by mitochondria after traumatic brain injury in the rat: Evidence for differentially sensitive populations in the cortex and hippocampus. J. Cereb. Blood Flow. Metab..

[151] Iannucci J., Dominy R., Bandopadhyay S., Arthur E.M., Noarbe B., Jullienne A., Krkasharyan M., Tobin R.P., Pereverzev A., Beevers S. (2024). Traumatic brain injury alters the effects of class II invariant peptide (CLIP) antagonism on chronic meningeal CLIP + B cells, neuropathology, and neurobehavioral impairment in 5xFAD mice. J. Neuroinflamm..

[152] Yu G.X., Mueller M., Hawkins B.E., Mathew B.P., Parsley M.A., Vergara L.A., Hellmich H.L., Prough D.S., Dewitt D.S. (2014). Traumatic brain injury in vivo and in vitro contributes to cerebral vascular dysfunction through impaired gap junction communication between vascular smooth muscle cells. J. Neurotrauma.

[153] Lin Y., Pan Y., Wang M., Huang X., Yin Y., Wang Y., Jia F., Xiong W., Zhang N., Jiang J.-y. (2012). Blood–brain barrier permeability is positively correlated with cerebral microvascular perfusion in the early fluid percussion-injured brain of the rat. Lab. Investig..

[154] Tanno H., Nockels R.P., Pitts L.H., Noble L.J. (1992). Breakdown of the Blood–Brain Barrier After Fluid Percussion Brain Injury in the Rat: Part 2: Effect of Hypoxia on Permeability to Plasma Proteins. J. Neurotrauma.

[155] Pitkanen A., Paananen T., Kyyriainen J., Das Gupta S., Heiskanen M., Vuokila N., Banuelos-Cabrera I., Lapinlampi N., Kajevu N., Andrade P. (2021). Biomarkers for posttraumatic epilepsy. Epilepsy Behav..

[156] Tobin R.P., Mukherjee S., Kain J.M., Rogers S.K., Henderson S.K., Motal H.L., Rogers M.K.N., Shapiro L.A. (2014). Traumatic brain injury causes selective, CD74-dependent peripheral lymphocyte activation that exacerbates neurodegeneration. Acta Neuropathol. Commun..

[157] Liu Y.-C., Tsai Y.-H., Tang S.-C., Liou H.-C., Kang K.-H., Liou H.-H., Jeng J.-S., Fu W.-M. (2018). Cytokine MIF Enhances Blood-Brain Barrier Permeability: Impact for Therapy in Ischemic Stroke. Sci. Rep..

[158] Galea I. (2021). The blood–brain barrier in systemic infection and inflammation. Cell. Mol. Immunol..

[159] Golub V.M., Reddy D.S. (2022). Post-Traumatic Epilepsy and Comorbidities: Advanced Models, Molecular Mechanisms, Biomarkers, and Novel Therapeutic Interventions. Pharmacol. Rev..

[160] Ivens S., Kaufer D., Flores L.P., Bechmann I., Zumsteg D., Tomkins O., Seiffert E., Heinemann U., Friedman A. (2007). TGF-beta receptor-mediated albumin uptake into astrocytes is involved in neocortical epileptogenesis. Brain.

[161] Bombardier C.H., Fann J.R., Temkin N.R., Esselman P.C., Barber J., Dikmen S.S. (2010). Rates of major depressive disorder and clinical outcomes following traumatic brain injury. Jama.

[162] Hart T., Hoffman J.M., Pretz C., Kennedy R., Clark A.N., Brenner L.A. (2012). A Longitudinal Study of Major and Minor Depression Following Traumatic Brain Injury. Arch. Phys. Med. Rehabil..

[163] Jorge R.E., Robinson R.G., Arndt S.V., Starkstein S.E., Forrester A.W., Geisler F. (1993). Depression following traumatic brain injury: A 1 year longitudinal study. J. Affect. Disord..

[164] Jorge R.E., Robinson R.G., Moser D., Tateno A., Crespo-Facorro B., Arndt S. (2004). Major depression following traumatic brain injury. Arch. Gen. Psychiatry.

[165] Mauri M.C., Paletta S., Colasanti A., Miserocchi G., Altamura A.C. (2014). Clinical and neuropsychological correlates of major depression following post-traumatic brain injury, a prospective study. Asian J. Psychiatry.

[166] Lavoie S., Sechrist S., Quach N., Ehsanian R., Duong T., Gotlib I.H., Isaac L. (2017). Depression in Men and Women One Year Following Traumatic Brain Injury (TBI): A TBI Model Systems Study. Front. Psychol..

[167] Bryant A.M., Rose N.B., Temkin N.R., Barber J.K., Manley G.T., McCrea M.A., Nelson L.D., Investigators T.-T. (2023). Profiles of Cognitive Functioning at 6 Months After Traumatic Brain Injury Among Patients in Level I Trauma Centers: A TRACK-TBI Study. JAMA Netw. Open.

[168] Lennon M.J., Brooker H., Creese B., Thayanandan T., Rigney G., Aarsland D., Hampshire A., Ballard C., Corbett A., Raymont V. (2023). Lifetime Traumatic Brain Injury and Cognitive Domain Deficits in Late Life: The PROTECT-TBI Cohort Study. J. Neurotrauma.

[169] Arciniegas D.B., Held K., Wagner P. (2002). Cognitive impairment following traumatic brain injury. Curr. Treat. Options Neurol..

[170] Wilson L., Horton L., Kunzmann K., Sahakian B.J., Newcombe V.F., Stamatakis E.A., von Steinbuechel N., Cunitz K., Covic A., Maas A. (2021). Understanding the relationship between cognitive performance and function in daily life after traumatic brain injury. J. Neurol. Neurosurg. Psychiatry.

[171] Wright T.J., Elliott T.R., Randolph K.M., Pyles R.B., Masel B.E., Urban R.J., Sheffield-Moore M. (2024). Prevalence of fatigue and cognitive impairment after traumatic brain injury. PLoS ONE.

[172] Zhu Y., Williams J., Beyene K., Trani J.F., Babulal G.M. (2024). Traumatic Brain Injury, Seizures, and Cognitive Impairment Among Older Adults. JAMA Netw. Open.

[173] Del Pozzo J., Spielman L., Yew B., Shpigel D.M., Selamanovic E., Dams-O’Connor K. (2024). Detecting and Predicting Cognitive Decline in Individuals with Traumatic Brain Injury: A Longitudinal Telephone-Based Study. J. Neurotrauma.

[174] Semple B.D., Zamani A., Rayner G., Shultz S.R., Jones N.C. (2019). Affective, neurocognitive and psychosocial disorders associated with traumatic brain injury and post-traumatic epilepsy. Neurobiol. Dis..

[175] Awan N., Weppner J., Kumar R.G., Juengst S.B., Dams-O’Connor K., Sevigny M., Zafonte R.D., Walker W.C., Szaflarski J.P., Wagner A.K. (2025). Impact of Post-Traumatic Epilepsy on Mental Health and Multidimensional Outcome and Quality of Life: An NIDILRR TBIMS Study. J. Neurotrauma.

[176] Scholten A.C., Haagsma J.A., Cnossen M.C., Olff M., van Beeck E.F., Polinder S. (2016). Prevalence of and Risk Factors for Anxiety and Depressive Disorders after Traumatic Brain Injury: A Systematic Review. J. Neurotrauma.

[177] Fann J.R., Katon W.J., Uomoto J.M., Esselman P.C. (1995). Psychiatric disorders and functional disability in outpatients with traumatic brain injuries. Am. J. Psychiatry.

[178] Osborn A.J., Mathias J.L., Fairweather-Schmidt A.K., Anstey K.J. (2017). Anxiety and comorbid depression following traumatic brain injury in a community-based sample of young, middle-aged and older adults. J. Affect. Disord..

[179] Stelfa G., Svalbe B., Vavers E., Duritis I., Dambrova M., Zvejniece L. (2022). Moderate traumatic brain injury triggers long-term risks for the development of peripheral pain sensitivity and depressive-like behavior in mice. Front. Neurol..

[180] Shultz S.R., MacFabe D.F., Foley K.A., Taylor R., Cain D.P. (2011). A single mild fluid percussion injury induces short-term behavioral and neuropathological changes in the Long–Evans rat: Support for an animal model of concussion. Behav. Brain Res..

[181] da Silva Fiorin F., do Espírito Santo C.C., do Nascimento R.S., França A.P., Royes L.F.F. (2024). Behavioral deficits after mild traumatic brain injury by fluid percussion in rats. Neurosci. Lett..

[182] Jones N.C., Cardamone L., Williams J.P., Salzberg M.R., Myers D., O’Brien T.J. (2008). Experimental traumatic brain injury induces a pervasive hyperanxious phenotype in rats. J. Neurotrauma.

[183] Shultz S.R., Bao F., Omana V., Chiu C., Brown A., Cain D.P. (2012). Repeated mild lateral fluid percussion brain injury in the rat causes cumulative long-term behavioral impairments, neuroinflammation, and cortical loss in an animal model of repeated concussion. J. Neurotrauma.

[184] Tan X.L., Wright D.K., Liu S., Hovens C., O’Brien T.J., Shultz S.R. (2016). Sodium selenate, a protein phosphatase 2A activator, mitigates hyperphosphorylated tau and improves repeated mild traumatic brain injury outcomes. Neuropharmacology.

[185] Shultz S.R., Bao F., Weaver L.C., Cain D.P., Brown A. (2013). Treatment with an anti-CD11d integrin antibody reduces neuroinflammation and improves outcome in a rat model of repeated concussion. J. Neuroinflamm..

[186] Fenn A.M., Gensel J.C., Huang Y., Popovich P.G., Lifshitz J., Godbout J.P. (2014). Immune activation promotes depression 1 month after diffuse brain injury: A role for primed microglia. Biol. Psychiatry.

[187] Lim S.W., Sung K.C., Shiue Y.L., Wang C.C., Chio C.C., Kuo J.R. (2017). Hyperbaric Oxygen Effects on Depression-Like Behavior and Neuroinflammation in Traumatic Brain Injury Rats. World Neurosurg..

[188] Levin E.D., Buccafusco J.J. (2006). Frontiers in Neuroscience. Animal Models of Cognitive Impairment.

[189] Lifshitz J., Witgen B., Grady M. (2007). Acute cognitive impairment after lateral fluid percussion brain injury recovers by 1 month: Evaluation by conditioned fear response. Behav. Brain Res..

[190] Spain A., Daumas S., Lifshitz J., Rhodes J., Andrews P.J., Horsburgh K., Fowler J.H. (2010). Mild Fluid Percussion Injury in Mice Produces Evolving Selective Axonal Pathology and Cognitive Deficits Relevant to Human Brain Injury. J. Neurotrauma.

[191] Gurkoff G.G., Gahan J.D., Ghiasvand R.T., Hunsaker M.R., Van K., Feng J.F., Shahlaie K., Berman R.F., Lyeth B.G., Folkerts M.M. (2013). Evaluation of metric, topological, and temporal ordering memory tasks after lateral fluid percussion injury. J. Neurotrauma.

[192] Fitzgerald J., Houle S., Cotter C., Zimomra Z., Martens K.M., Vonder Haar C., Kokiko-Cochran O.N. (2022). Lateral Fluid Percussion Injury Causes Sex-Specific Deficits in Anterograde but Not Retrograde Memory. Front. Behav. Neurosci..

[193] Newell E.A., Todd B.P., Luo Z., Evans L.P., Ferguson P.J., Bassuk A.G. (2020). A Mouse Model for Juvenile, Lateral Fluid Percussion Brain Injury Reveals Sex-Dependent Differences in Neuroinflammation and Functional Recovery. J. Neurotrauma.

[194] Schurman L.D., Smith T.L., Morales A.J., Lee N.N., Reeves T.M., Phillips L.L., Lichtman A.H. (2017). Investigation of left and right lateral fluid percussion injury in C57BL6/J mice: In vivo functional consequences. Neurosci. Lett..

[195] Hetzer S.M., Casagrande A., Qu’d D., Dobrozsi N., Bohnert J., Biguma V., Evanson N.K., McGuire J.L., Hetzer S.M., Casagrande A. (2023). Early Measures of TBI Severity Poorly Predict Later Individual Impairment in a Rat Fluid Percussion Model. Brain Sci..

[196] Gurkoff G.G., Giza C.C., Hovda D.A. (2006). Lateral fluid percussion injury in the developing rat causes an acute, mild behavioral dysfunction in the absence of significant cell death. Brain Res..

[197] Hylin M.J., Orsi S.A., Zhao J., Bockhorst K., Perez A., Moore A.N., Dash P.K. (2013). Behavioral and histopathological alterations resulting from mild fluid percussion injury. J. Neurotrauma.

[198] Ballard C., Gauthier S., Corbett A., Brayne C., Aarsland D., Jones E. (2011). Alzheimer’s disease. Lancet.

[199] Gallardo G., Holtzman D.M., Takashima A., Wolozin B., Buee L. (2019). Amyloid-β and Tau at the Crossroads of Alzheimer’s Disease. Tau Biology.

[200] Mielke M.M., Ransom J.E., Mandrekar J., Turcano P., Savica R., Brown A.W. (2022). Traumatic Brain Injury and Risk of Alzheimer’s Disease and Related Dementias in the Population. J. Alzheimer’s Dis..

[201] Schaffert J., LoBue C., White C.L., Chiang H.S., Didehbani N., Lacritz L., Rossetti H., Dieppa M., Hart J., Cullum C.M. (2018). Traumatic brain injury history is associated with an earlier age of dementia onset in autopsy-confirmed Alzheimer’s disease. Neuropsychology.

[202] Gardner R.C., Burke J.F., Nettiksimmons J., Kaup A., Barnes D.E., Yaffe K. (2014). Dementia risk after traumatic brain injury vs nonbrain trauma: The role of age and severity. JAMA Neurol..

[203] Muroni A., Floris G., Borghero G., Ardu S., Pateri M.I., Pilotto S., Pisano G., Defazio G. (2024). Point prevalence of epilepsy in dementia: A “real-world” estimate. Epileptic Disord..

[204] Xu Y., Lavrencic L., Radford K., Booth A., Yoshimura S., Anstey K.J., Anderson C.S., Peters R. (2021). Systematic review of coexistent epileptic seizures and Alzheimer’s disease: Incidence and prevalence. J. Am. Geriatr. Soc..

[205] Subota A., Pham T., Jetté N., Sauro K., Lorenzetti D., Holroyd-Leduc J. (2017). The association between dementia and epilepsy: A systematic review and meta-analysis. Epilepsia.

[206] Leitner D., Pires G., Kavanagh T., Kanshin E., Askenazi M., Ueberheide B., Devinsky O., Wisniewski T., Drummond E. (2024). Similar brain proteomic signatures in Alzheimer’s disease and epilepsy. Acta Neuropathol..

[207] Martin S.P., Leeman-Markowski B.A. (2023). Proposed mechanisms of tau: Relationships to traumatic brain injury, Alzheimer’s disease, and epilepsy. Front. Neurol..

[208] Toscano E.C.B., Vieira É.L.M., Grinberg L.T., Rocha N.P., Brant J.A.S., Paradela R.S., Giannetti A.V., Suemoto C.K., Leite R.E.P., Nitrini R. (2023). Hyperphosphorylated Tau in Mesial Temporal Lobe Epilepsy: A Neuropathological and Cognitive Study. Mol. Neurobiol..

[209] Hawkins B.E., Krishnamurthy S., Castillo-Carranza D.L., Sengupta U., Prough D.S., Jackson G.R., DeWitt D.S., Kayed R. (2013). Rapid Accumulation of Endogenous Tau Oligomers in a Rat Model of Traumatic Brain Injury: Possible Link Between Traumatic Brain Injury and Sporadic Tauopathies *. J. Biol. Chem..

[210] Altuna M., Olmedo-Saura G., Carmona-Iragui M., Fortea J. (2022). Mechanisms Involved in Epileptogenesis in Alzheimer’s Disease and Their Therapeutic Implications. Int. J. Mol. Sci..

[211] Vicente M., Addo-Osafo K., Vossel K. (2024). Latest advances in mechanisms of epileptic activity in Alzheimer’s disease and dementia with Lewy Bodies. Front. Neurol..

[212] Paudel Y.N., Angelopoulou E., Jones N.C., O’Brien T.J., Kwan P., Piperi C., Othman I., Shaikh M.F. (2019). Tau Related Pathways as a Connecting Link between Epilepsy and Alzheimer’s Disease. ACS Chem. Neurosci..

[213] Hoshino S., Tamaoka A., Takahashi M., Kobayashi S., Furukawa T., Oaki Y., Mori O., Matsuno S., Shoji S., Inomata M. (1998). Emergence of immunoreactivities for phosphorylated tau and amyloid-beta protein in chronic stage of fluid percussion injury in rat brain. Neuroreport.

[214] Iwata A., Chen X.H., McIntosh T.K., Browne K.D., Smith D.H. (2002). Long-term accumulation of amyloid-beta in axons following brain trauma without persistent upregulation of amyloid precursor protein genes. J. Neuropathol. Exp. Neurol..

[215] Kang J.Q. (2021). Epileptic Mechanisms Shared by Alzheimer’s Disease: Viewed via the Unique Lens of Genetic Epilepsy. Int. J. Mol. Sci..

[216] Texidó L., Martín-Satué M., Alberdi E., Solsona C., Matute C. (2011). Amyloid β peptide oligomers directly activate NMDA receptors. Cell Calcium.

[217] Gu Z., Liu W., Yan Z. (2009). {beta}-Amyloid impairs AMPA receptor trafficking and function by reducing Ca2+/calmodulin-dependent protein kinase II synaptic distribution. J. Biol. Chem..

[218] Zhang Y., Guo O., Huo Y., Wang G., Man H.Y. (2018). Amyloid-β Induces AMPA Receptor Ubiquitination and Degradation in Primary Neurons and Human Brains of Alzheimer’s Disease. J. Alzheimer’s Dis..

[219] Freeman L.C., Ting J.P. (2016). The pathogenic role of the inflammasome in neurodegenerative diseases. J. Neurochem..

[220] Johnson N.H., de Rivero Vaccari J.P., Bramlett H.M., Keane R.W., Dietrich W.D. (2023). Inflammasome activation in traumatic brain injury and Alzheimer’s disease. Transl. Res..

[221] Li Q., Zhao P., Wen Y., Zou Z., Qin X., Tan H., Gong J., Wu Q., Zheng C., Zhang K. (2023). Polydatin ameliorates traumatic brain injury-induced secondary brain injury by inhibiting nlrp3-induced neuroinflammation associated with SOD2 acetylation. Shock.

[222] de Rivero Vaccari J.P., Lotocki G., Alonso O.F., Bramlett H.M., Dietrich W.D., Keane R.W. (2009). Therapeutic neutralization of the NLRP1 inflammasome reduces the innate immune response and improves histopathology after traumatic brain injury. J. Cereb. Blood Flow. Metab..

[223] Guerreiro R., Wojtas A., Bras J., Carrasquillo M., Rogaeva E., Majounie E., Cruchaga C., Sassi C., Kauwe J.S., Younkin S. (2013). TREM2 variants in Alzheimer’s disease. N. Engl. J. Med..

[224] Saber M., Kokiko-Cochran O., Puntambekar S.S., Lathia J.D., Lamb B.T. (2017). Triggering Receptor Expressed on Myeloid Cells 2 Deficiency Alters Acute Macrophage Distribution and Improves Recovery after Traumatic Brain Injury. J. Neurotrauma.

[225] Gyoneva S., Kim D., Katsumoto A., Kokiko-Cochran O.N., Lamb B.T., Ransohoff R.M. (2015). Ccr2 deletion dissociates cavity size and tau pathology after mild traumatic brain injury. J. Neuroinflamm..

[226] Popiolek-Barczyk K., Ciechanowska A., Ciapała K., Pawlik K., Oggioni M., Mercurio D., De Simoni M.G., Mika J. (2020). The CCL2/CCL7/CCL12/CCR2 pathway is substantially and persistently upregulated in mice after traumatic brain injury, and CCL2 modulates the complement system in microglia. Mol. Cell Probes.

[227] Puntambekar S.S., Saber M., Lamb B.T., Kokiko-Cochran O.N. (2018). Cellular players that shape evolving pathology and neurodegeneration following traumatic brain injury. Brain Behav. Immun..

[228] Morganti J.M., Jopson T.D., Liu S., Riparip L.K., Guandique C.K., Gupta N., Ferguson A.R., Rosi S. (2015). CCR2 antagonism alters brain macrophage polarization and ameliorates cognitive dysfunction induced by traumatic brain injury. J. Neurosci..

[229] Garga N., Lowenstein D.H. (2006). Posttraumatic epilepsy: A major problem in desperate need of major advances. Epilepsy Curr..

[230] Al-Beltagi M., Saeed N.K. (2022). Epilepsy and the gut: Perpetrator or victim?. World J. Gastrointest. Pathophysiol..

[231] Bansal V., Costantini T., Kroll L., Peterson C., Loomis W., Eliceiri B., Baird A., Wolf P., Coimbra R. (2009). Traumatic brain injury and intestinal dysfunction: Uncovering the neuro-enteric axis. J. Neurotrauma.

[232] Katzenberger R.J., Ganetzky B., Wassarman D.A. (2015). The gut reaction to traumatic brain injury. Fly.

[233] Zhu C.S., Grandhi R., Patterson T.T., Nicholson S.E. (2018). A Review of Traumatic Brain Injury and the Gut Microbiome: Insights into Novel Mechanisms of Secondary Brain Injury and Promising Targets for Neuroprotection. Brain Sci..

[234] Venkatasamy L., Iannucci J., Pereverzev A., Hoar J., Huber E., Ifegbo A., Dominy R., El-Hakim Y., Mani K.K., Dabney A. (2025). Systemic IGF-1 administration prevents traumatic brain injury induced gut permeability, dysmorphia, dysbiosis, and the increased number of immature dentate granule cells. Acta Neuropathol. Commun..

[235] Ma E.L., Smith A.D., Desai N., Cheung L., Hanscom M., Stoica B.A., Loane D.J., Shea-Donohue T., Faden A.I. (2017). Bidirectional brain-gut interactions and chronic pathological changes after traumatic brain injury in mice. Brain Behav. Immun..

[236] Hanscom M., Loane D.J., Shea-Donohue T. (2021). Brain-gut axis dysfunction in the pathogenesis of traumatic brain injury. J. Clin. Investig..

[237] Shi D., Niu Z., Li L., Wang L., Ren S., Li J., Wu G. (2026). Neuroinflammation promotes the development of pharmacoresistant epilepsy by triggering the MSK1/CREB signaling pathway. Sci. Rep..

[238] De Caro C., Leo A., Nesci V., Ghelardini C., di Cesare Mannelli L., Striano P., Avagliano C., Calignano A., Mainardi P., Constanti A. (2019). Intestinal inflammation increases convulsant activity and reduces antiepileptic drug efficacy in a mouse model of epilepsy. Sci. Rep..

[239] Suleymanova E., Karan A. (2025). The Role of Blood-Brain Barrier Disruption in Epilepsy: Mechanisms and Consequences. Neurol. Int..

[240] Dadas A., Janigro D. (2019). Breakdown of blood brain barrier as a mechanism of post-traumatic epilepsy. Neurobiol. Dis..

[241] Peng X., Luo Z., He S., Zhang L., Li Y. (2021). Blood-Brain Barrier Disruption by Lipopolysaccharide and Sepsis-Associated Encephalopathy. Front. Cell. Infect. Microbiol..

[242] Hu A., Yuan H., Qin Y., Zhu Y., Zhang L., Chen Q., Wu L. (2022). Lipopolysaccharide (LPS) increases susceptibility to epilepsy via interleukin-1 type 1 receptor signaling. Brain Res..

[243] Auvin S., Shin D., Mazarati A., Sankar R. (2010). Inflammation induced by LPS enhances epileptogenesis in immature rat and may be partially reversed by IL1RA. Epilepsia.

[244] Ndode-Ekane X.E., Ali I., Santana-Gomez C., Andrade P., Immonen R., Casillas-Espinosa P., Paananen T., Manninen E., Puhakka N., Smith G. (2024). Epilepsy phenotype and its reproducibility after lateral fluid percussion-induced traumatic brain injury in rats: Multicenter EpiBioS4Rx study project 1. Epilepsia.

[245] Heiskanen M., Ndode-Ekane X.E., Ali I., Santana-Gomez C., Puhakka N., Gupta S.D., Andrade P., Immonen R., Casillas-Espinosa P., Manninen E. (2025). Plasma microRNAs as prognostic biomarkers for development of severe epilepsy after experimental traumatic brain injury-EpiBioS4Rx Project 1 study. Epilepsia.

[246] Morales-Villagrán A., Salazar-Sánchez J.C., Chiprés-Tinajero G.A., Medina-Ceja L., Ortega-Ibarra J. (2023). A novel hydro-pneumatic fluid percussion device for inducing traumatic brain injury: Assessment of sensory, motor, cognitive, molecular, and morphological outcomes in rodents. Front. Mol. Neurosci..

[247] Sharma A., Cabeen R., Harris N.G., Wright D.K., Tohka J., Grohn O., Bennett A., Alba C., Bhagavatula S., Garner R. (2025). Bundle-specific tractography approach for identifying white matter microstructural changes following traumatic brain injury in rats: An EpiBioS4Rx study. Imaging Neurosci..

[248] Heiskanen M., Jääskeläinen O., Manninen E., Das Gupta S., Andrade P., Ciszek R., Gröhn O., Herukka S.-K., Puhakka N., Pitkänen A. (2022). Plasma Neurofilament Light Chain (NF-L) Is a Prognostic Biomarker for Cortical Damage Evolution but Not for Cognitive Impairment or Epileptogenesis Following Experimental TBI. Int. J. Mol. Sci..

[249] Das M., Leonardo C.C., Rangooni S., Pennypacker K.R., Mohapatra S., Mohapatra S.S. (2011). Lateral fluid percussion injury of the brain induces CCL20 inflammatory chemokine expression in rats. J. Neuroinflamm..

[250] Manninen E., Chary K., Lapinlampi N., Andrade P., Paananen T., Sierra A., Tohka J., Gröhn O., Pitkänen A. (2021). Acute thalamic damage as a prognostic biomarker for post-traumatic epileptogenesis. Epilepsia.

[251] Manninen E., Cabeen R., Immonen R., Ndode-Ekane X.E., Andrade P., Harris N.G., Wright D.K., Tohka J., Gröhn O., Pitkänen A. (2025). Damage in thalamic projection to perilesional cortex as a prognostic biomarker for experimental post-traumatic epilepsy. Epilepsia.

[252] Ondek K., Pevzner A., Tercovich K., Schedlbauer A.M., Izadi A., Ekstrom A.D., Cowen S.L., Shahlaie K., Gurkoff G.G. (2020). Recovery of Theta Frequency Oscillations in Rats Following Lateral Fluid Percussion Corresponds With a Mild Cognitive Phenotype. Front. Neurol..

[253] Xiong Y., Mahmood A., Chopp M. (2013). Animal models of traumatic brain injury. Nat. Rev. Neurosci..

[254] Peterson T.C., Maass W.R., Anderson J.R., Anderson G.D., Hoane M.R. (2015). A behavioral and histological comparison of fluid percussion injury and controlled cortical impact injury to the rat sensorimotor cortex. Behav. Brain Res..

[255] Echegoyen J., Armstrong C., Morgan R.J., Soltesz I. (2009). Single application of a CB1 receptor antagonist rapidly following head injury prevents long-term hyperexcitability in a rat model. Epilepsy Res..

[256] Gurkoff G.G., Giza C.C., Shin D., Auvin S., Sankar R., Hovda D.A. (2009). Acute neuroprotection to pilocarpine-induced seizures is not sustained after traumatic brain injury in the developing rat. Neuroscience.

[257] Reddy D.S., Golub V.M., Ramakrishnan S., Churn S.B., Shapiro L.A., Iannucci J., Pitkänen A., Galanopoulou A.S., Maganti R., Boison D. (2025). Advances and challenges in experimental models of posttraumatic epilepsy for therapeutic interventions. Pharmacol. Rev..

